# Investigation of the Circular Transcriptome in Alzheimer’s Disease Brain

**DOI:** 10.1007/s12031-024-02236-0

**Published:** 2024-07-09

**Authors:** Yulan Gao, Si-Mei Xu, Yuning Cheng, Konii Takenaka, Grace Lindner, Michael Janitz

**Affiliations:** School of Biotechnology and Biomolecular Sciences, University of New South Wales, Sydney, Australia

**Keywords:** circRNAs, Transcriptome, RNA sequencing, Alzheimer’s disease, Human brain

## Abstract

Circular RNAs (circRNAs) are a subclass of non-coding RNAs which have demonstrated potential as biomarkers for Alzheimer’s disease (AD). In this study, we conducted a comprehensive exploration of the circRNA transcriptome within AD brain tissues. Specifically, we assessed circRNA expression patterns in the dorsolateral prefrontal cortex collected from nine AD-afflicted individuals and eight healthy controls. Utilising two circRNA detection tools, CIRI2 and CIRCexplorer2, we detected thousands of circRNAs and performed a differential expression analysis. CircRNAs which exhibited statistically significantly differential expression were identified as AD-specific differentially expressed circRNAs. Notably, our investigation revealed 120 circRNAs with significant upregulation and 1325 circRNAs displaying significant downregulation in AD brains when compared to healthy brain tissue. Additionally, we explored the expression profiles of the linear RNA counterparts corresponding to differentially expressed circRNAs in AD-afflicted brains and discovered that the linear RNA counterparts exhibited no significant changes in the levels of expression. We used CRAFT tool to predict that circUBE4B had potential to target miRNA named as hsa-miR-325-5p, ultimately regulated *CD44* gene. This study provides a comprehensive overview of differentially expressed circRNAs in the context of AD brains, underscoring their potential as molecular biomarkers for AD. These findings significantly enhance our comprehension of AD’s underlying pathophysiological mechanisms, offering promising avenues for future diagnostic and therapeutic developments.

**Supplementary Information:**

The online version contains supplementary material available at 10.1007/s12031-024-02236-0.

## Introduction

Alzheimer’s disease (AD) is a neurodegenerative disease, classified based on its progressive decline and loss of neurons (Tom SE et al. 2015). As one of the main causes of dementia, AD has two categories, namely early-onset or sporadic AD that occurs after the age of 65, and familial AD that contains autosomal dominant missense mutations (Bhole RP et al. 2024). In particular, dysfunction of the dorsolateral prefrontal cortex is associated with impaired executive control function and working memory (Hertrich I et al. 2021; Kumar S et al. 2017) The AD pathology primarily involves misfolded, oligomerised amyloid β (Aβ) plaques (Grundke-Iqbal I et al. 1986) and accumulated neurofibrillary tangles (NFTs) (Glenner GG and Wong CW 1984), which are assessed for in AD pathology progression (Braak CD, E. Braak and F. Piette 1992). Despite ongoing research, the knowledge of AD-associated transcripts and the relationship between their dysfunction and the pathological mechanism is still elusive. Hence, a transcriptomic study on that investigates the effect of related genes and transcripts in AD has become crucial (Annese A et al. 2018; Bagyinszky E et al. 2020). Neurofilament light (NfL) and glial fibrillary acidic protein (GFAP) are proteins that have also been disclosed as imperative biomarker candidates in AD pathology (Benedet AL et al. 2021; Mantellatto G M et al. 2024; Pereira JB et al. 2021; Zhao Y et al. 2019). Remarkably, NfL is associated with the apolipoprotein E (APOE ε4) allele which is a significant biomarker in the identification of familial AD (Hawley NA et al. 2023). GFAP correlates with Aβ plaques and acts as a marker of astrogliosis in AD brain (O'Connor A et al. 2023). Moreover, advanced technology has unveiled that mutations of heterogenous nuclear ribonucleoproteins (hnRNPs) might play a crucial role as a regulatory factor in AD (Bartolomé-Nafría A et al. 2024). For instance, mutations on the prion-like domains of low complexity of hnRNPs were identified to exhibit association of amyloid fibril with neurodegenerative disease through accumulating toxicity to disorder (Lim L et al. 2016). TDP-43, a protein released in neuronal cells, was observed with features of hyperphosphorylation and ubiquitination in the progression of AD during synaptic and cognitive deterioration (Gao F et al. 2022; Lim L et al. 2016; Mantellatto G M et al. 2024) Transcriptome profiling can unravel pathogenesis mechanisms including neuronal transports, amyloid precursor protein (APP) synthesis and protein interaction during different onset stages of AD.

Circular RNAs (circRNAs) are non-coding RNAs (ncRNAs) and comprise of a covalently closed loop structure derived from the back-splicing of the pre-mRNA. Back-splicing is a spliceosome-dependent activity responsible for the upstream 5′-donor site ligating to the downstream 3′-acceptor site of the target exons (Jeck WR and Sharpless NE 2014; Wilusz JE and Sharp PA 2013). The ligated part is classified as back-splice junction (BSJ). The distinctive and intrinsic feature of circRNAs is the lack of a 5′ cap and 3′ poly (A) tail which is subsequent for their high stability and longer half-life compared to linear RNAs (Jeck WR and Sharpless NE 2014).

CircRNAs have multiple functions such as gene expression regulatory potential, translational capacity, microRNA (miRNA) sponging (Hansen TB et al. 2013), protein interaction (Goodrich JA and Kugel JF 2006) and competition with linear RNAs production (Ashwal-Fluss R et al. 2014). A significant number of circRNAs enriched in the human brain has been observed in past studies (Jeck WR and Sharpless NE 2014; Rybak-Wolf A et al. 2015; Zimmerman AJ et al. 2020). These features preposition circRNAs as disease biomarkers for detection and monitoring pathogenesis of AD (Huang J-L et al. 2020; Meng S et al. 2017). Previous studies (Lukiw W et al. 2016; Lukiw WJ et al. 2015; Shi Z et al. 2017) have demonstrated that the expression of brain tissue-specific circRNAs promotes multiple downstream responses including amyloid β (Aβ) generation and clearance, neuroinflammation, neuronal oxidative stress and autophagy. A study by Lukiw WJ et al. (2015) revealed that deficient levels ciRS-7 is ensued with failure to compete with miRNA-7 as a sponging effect, which can lead to higher expression of miRNA-7 and subsequent downregulation of the ubiquitin-conjugating enzyme UBE2A responsible for Aβ clearance. ciRS-7 also plays a role in neuroprotection through promoting proteasome and lysosome for degradation of APP and beta-site APP cleaving enzyme-1 (BACE1) levels (Shi Z et al. 2017). Neuroinflammation can be induced via the interaction of circPTK2 and miR-29b by activating microglial cells (González-Scarano F and Baltuch G, 1999; Shi Z et al. 2017). Dysregulation of circCwc27 regulates the binding affinity of purine-rich element-binding protein A (Pur-⍺) to the promoter of APP, which further control the level of cognitive decline in AD (Song C et al. 2022). To discriminate autosomal-dominant AD from sporadic AD, circPSEN1 was detected from in silico analysis which suggests its regulatory potentials in AD pathogenesis pathway and neuroinflammation (Chen HH et al. 2022). CircHomer1a, expressed in the dorsolateral prefrontal cortex, was found to play a role in synaptic expression and AD-related cognitive dysfunction (Dube U *et al.*, 2019; Zimmerman AJ et al. 2020).

Despite these advances, what is understood about circRNAs’ role in the pathology of neurodegeneration is limited and entails further research. Here, we investigate the circular transcriptome in the AD brain, aiming towards the identification of AD-specific circRNAs.

## Materials and Methods

### Accession and Quality Check of RNA Sequence Data and Library Preparation of circRNAs

For this study, total RNA sequencing (RNA-seq) data in FASTQ format, derived from dorsolateral prefrontal cortex, were downloaded from the NCBI database (accession number GSE53697). The full details of sequencing data generation steps can be found in Scheckel C et al. (2016)’s study. In summary, the control cohort was selected based on the individual’s exhibition of AD pathology in the form of neurofibrillary tangles and plaques. The AD samples were selected based on a clinical dementia rating (CDR) between four and five, with short post-mortem intervals (PMI) (Scheckel C et al. 2016). The sample tissues were subjected to Trizol (Invitrogen) extraction and the RNA templates were prepared following the Illumina high-throughput TruSeq RNA sample preparation guidelines. There have been no enriched circRNAs at the performance of ribosomal RNA (rRNA) depletion. As described in the source paper (Scheckel C et al. 2016), following rRNA depletion and DNase treatment on the brain samples and neuroblastoma cell lines, AD samples 7–9 and control samples 6–8 in this study were sequenced using Illumina HiSeq 2500 at the New York Genome Centre which yielded 125-bp paired end reads, while the remaining subjects were sequenced on an Illumina HiSeq 2000 system at the Rockefeller University Genomics Resource Centre, which produced 100-bp paired end reads. A quality check on the raw sequence data files was performed using FastQC v0.11.9 (Andrews S, 2010) to ensure that the FASTQ files generated reads of sufficient quality. Adaptors were removed using Trimmomatic v0.39 (Bolger AM et al. 2014) and the quality of the trimmed data files was also evaluated with FastQC prior to the circRNA detection pipeline (Andrews S, 2010).

### circRNAs Alignment and Identification

Read alignment and detection of circRNAs were performed using two different workflows with different tools and parameters. Both methods required an alignment to the UCSC hg38 reference genome obtained from the GENCODE v43 (GRCh38.p13) annotation file. The CIRI2 pathway utilises the Burrows-Wheeler alignment (BWA)-MEM v0.7.17 algorithm to align the query data (Li H and Durbin R, 2009) and produce Sequence Alignment Map (SAM) files. The SAM files containing the mapped sequences were processed through CIRI2 v2.0.6 (circRNA identifier) (Gao Y et al. 2015; Gao Y et al. 2018) for circRNA identification and annotation. The CIRCexplorer2 pathway began with Spliced Transcripts Alignment to a Reference (STAR) v2.7.6a (Dobin A et al. 2012) to generate Chimeric.out.junction files, followed by the CIRCexplorer2 pipeline (Zhang XO et al. 2016) for circRNA identification and annotation. These files were initially parsed using the CIRCexplorer2 parse module to analyse and pack the back-splice junction (BSJ) information into Browser Extensible Data (BED) files that were previously annotated by CIRCexplorer2’s annotate function. The output circRNA data from both CIRI2 and CIRCexplorer2 were selected using the margin of BSJ reads greater or equal to two, to reduce the likelihood of false positives. The common circRNAs detected by both circRNA detection tools were merged into a single file to remove redundant circRNAs, which would further reduce the likelihood of false positives.

### Linear RNA Analytical Pipeline

HISAT2 v2.2.0 (Kim D et al. 2019) was used to align the trimmed data to the genome index built on the reference genome UCSC hg38 using the HISAT build module. In order to assemble and quantify the linear transcripts, SAMtools v1.15.1 (Danecek P et al. 2021) was utilised to convert the HISAT2 output file format from SAM files to Binary Alignment Map (BAM) files, followed by converting the assembled transcripts into Gene Transfer Format (GTF) files from the StringTie v1.3.4d algorithm (Pertea M et al. 2015). A non-redundant set of transcripts was generated regarding the GTF files using the StringTie merge module.

### Differential Expression Analysis

Circular RNAs. The annotated circRNA information was analysed in R environment v4.2.2 (http://www.R-project.org/) using edgeR v3.40.2 (Robinson MD et al. 2010), limma v3.54.2 (Phipson B et al. 2016), dplyr v1.1.2 (Wickham H FR, Henry L, Müller K, Vaughan D 2023) and tidyverse v2.0.0 (Wickham H VD, Girlich M, 2023). Average expression values in the merged data files were calculated using the counts per million (CPM) mapped reads as the unit. The library size from common circRNA read counts was normalised from a calculated normalised factor using trimmed mean of *M*-values (TMM). In addition to differential expression analysis on circRNAs, library size-normalised data were fit into linear model for series of arrays, followed by applying empirical Bayes statistics for differential expression onto circRNAs which ranked the order of circRNAs by evidence of arbitrary number of contrasts. All differentially expressed circRNAs that exhibited statistical significance (false discovery rate unadjusted *p*-value < 0.05) were selected. The *p*-value was later adjusted to a more stringent threshold of less than 0.1 using Banjamini-Hochberg procedure via limma (Phipson B et al. 2016) to acquire a larger number of differentially expressed circRNAs for a more comprehensive coverage. In order to visualise the differential expression of circRNAs through a robust volcano plot, the ggplot2 v3.4.2 package (H W, 2016) in R was used to display the up- and downregulated, as well as stable circRNAs.

Linear RNAs. In preparation for generating a linear transcript count matrix used for differential expression analysis, the abundance of gene read counts output from StringTie—eB arguments were supplied to the developer’s Python v3 script (Shumate A et al. 2022). The gene count matrix was then imported into the R environment for differential expression analysis on linear transcripts. Average expression values for linear RNAs were also calculated using CPM as the unit. The same packages and procedures used in the differentially expressed circRNAs analysis were also used for linear differential expression analysis (i.e. unadjusted *p* < 0.05) and volcano plots visualisation.

### Data Visualisation

To visualise and compare the data, boxplots exhibiting CPM values from control and AD were constructed using Prism 9 v9.4.1 (https://www.graphpad.com/). The CPM values of the top ten upregulated circRNAs and their linear counterparts in AD samples were compared with control samples. The selection of upregulated circRNAs were then visualised on CircView (Feng J et al. 2017) to show contributing exons and genomic loci coordinates.

### Gene Ontology Enrichment and KEGG Enrichment Analysis

The R package, clusterProfiler (Wu T et al. 2021; Yu G et al. 2012) was used for the analysis of Gene Ontology (GO) enrichment from biological processes and cellular component aspects. The input gene dataset included the top 500 differentially expressed linear RNAs and downregulated circRNAs for consistency and more comprehensive enrichment analysis, and 120 differentially expressed upregulated circRNAs. The GO over-representation test utilised the Benjamini and Hochberg (BH) *p-*value adjustment method to reduce false discovery rate for multiple comparison (Benjamini Y and Hochberg Y, 1995). Kyoto Encycopedia of Gene and Genomes (KEGG) enrichment analysis was performed for upregulated and top 500 downregulated circRNAs using DAVID (https://david.ncifcrf.gov/tools.jsp) (Huang da W et al. 2009; Sherman BT et al. 2022). The results of the enriched KEGG terms from DAVID were used to construct an enrichment bubble through SRplot (https://www.bioinformatics.com.cn/srplot) with a *p*-value cut-off of 0.5 (Tang D et al. 2023).

### CircRNA-miRNA-mRNA Network

The BSJ coordinates of the selected circRNAs between CIRI2, CIRCexplorer2 and CircView outputs were validated against each other. Ten of these selected significantly upregulated circRNAs in AD samples were subjected to explore the potential miRNA-binding sites using the CRAFT v1.0 software and pipeline (Dal Molin A et al*.*, 2022). Default parameters were used for functional analysis, table generation and graphical output. Information regarding the circRNA host gene, miRNA and target gene were retrieved from the CRAFT results. The circRNA-miRNA-mRNA network was established for selected circRNAs that exhibited several miRNA-binding sites greater or equal to seven.

## Results

### Circular Transcriptome Sequencing Metrics

The fastQC output revealed an average GC content of 43.39% for the nine AD samples and 44.25% for the eight control samples (Table S1). Across all trimmed samples, an average of 47,334,570 reads and 44,769,873 was detected with > 80% alignment rate for the nine AD samples and eight control samples, respectively. The output files from CIRI2 and CIRCexplorer2 were merged to identify the common circRNAs, with the average number of circRNAs detected in AD and control samples being 4676 and 5132, respectively (Table S1).

### Differential circRNA Expression

A comparative analysis of circRNA expression between AD and healthy brain tissue revealed nine downregulated circRNAs using an adjusted *p*-value < 0.1 (FigA). There were 120 upregulated and 1325 downregulated circRNAs (*p*-value < 0.05) in the AD brain as depicted in FigB and listed in Table S2. The result of differential expression analysis for their linear counterparts is shown in Fig. S2.

**Fig. 1:**
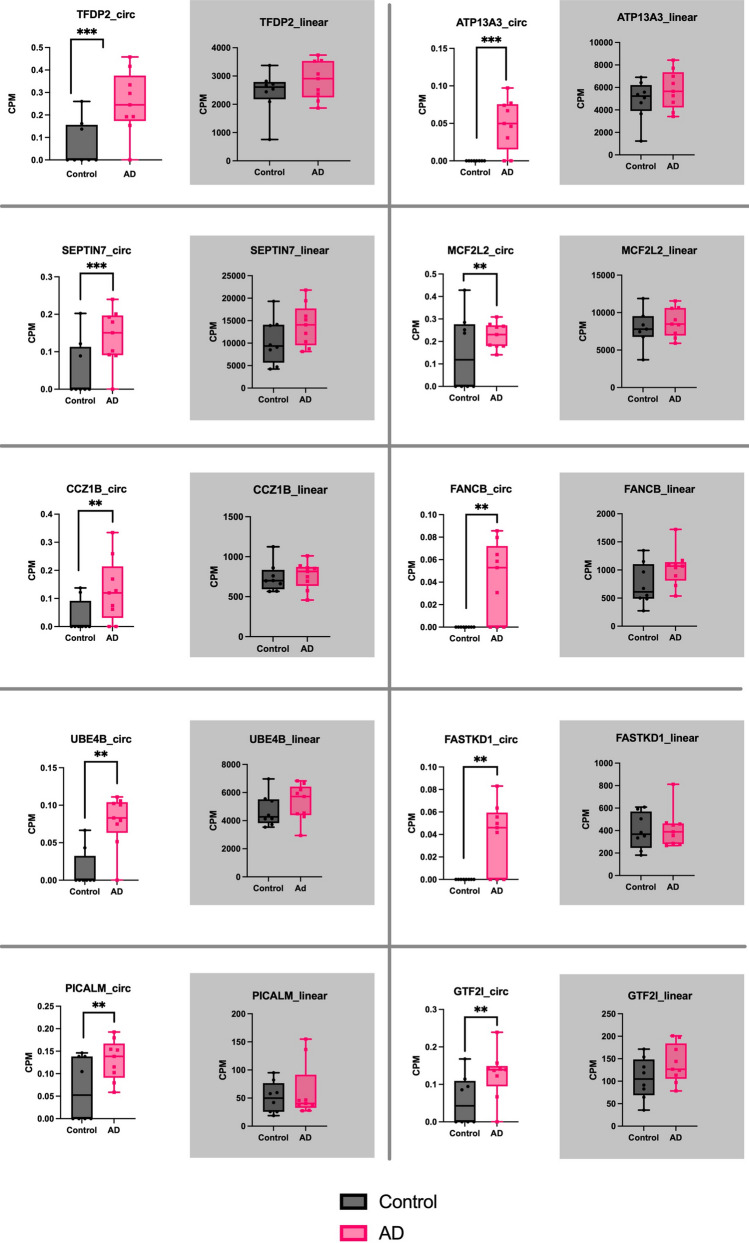
Top 10 differentially expressed circRNAs (*p*-value < 0.05) and their linear counterparts. The number of asterisks represents the level of significance of the CPM value in AD comparing to control samples (***p-value < 0.001, **p-value < 0.01). None of the linear counterparts were statistically significant. The control and AD samples were presented in grey and pink colours, respectively. The relative linear counterparts include a grey background. The plots were created using Prism v9.4.1

The top ten upregulated circRNAs by the smallest p-value were selected for further analysis (Table 1). The BSJ coordinates of these circRNAs matched between CIRI2, CIRCexplorer2 and CircView tools, confirming their proper genomic annotation. The log-fold change (FC) values for the selected upregulated circRNAs were all greater than 1.5. Boxplots were generated according to the CPM values for each selected circRNAs and their corresponding linear transcripts (Fig. 1). In comparison, the linear counterparts had no significantly differential expression between AD and control samples (Fig. 1 and Table S3). Of note, circATP13A3, circFANCB and circFASTKD1 presented an average CPM value of zero in the healthy control brain samples (Fig. 1). These circRNAs were considered uniquely expressed in AD brains.

**Table 1:** Top ten circRNAs most upregulated in AD

Host gene	BSJ genomic coordinates	CPM^1^ AD	CPM control^2^	logFC^3^	*p*-value	Number of reads^4^
TFDP2	chr3:142054115–142101841	0.254	0.070	2.36	0.0001	59
ATP13A3	chr3:194459471–194462236	0.049	0.000	1.85	0.0005	7
SEPTIN7	chr7:35879823–35885879	0.139	0.050	1.96	0.0007	50
MCF2L2	chr3:183379297–183389779	0.226	0.150	1.76	0.0020	96
CCZ1B	chr7:6814764–6822364	0.127	0.032	1.83	0.0022	74
FANCB	chrX:14843660–14865580	0.041	0.000	1.62	0.0023	17
UBE4B	chr1:10149184–10161286	0.079	0.014	1.74	0.0023	33
FASTKD1	chr2:169538013–169544835	0.038	0.000	1.53	0.0042	14
PICALM	chr11:86012281–86031611	0.130	0.066	1.55	0.0057	57
GTF2I	chr7:74705164–74718941	0.127	0.058	1.58	0.0059	53

^1^*CPM* back-splice junction

^2^*CPM* average counts per million

^3^*logFC* log-fold change

^4^Number of reads, sum of number of reads across all samples

### Visualisation of circRNAs

**Fig. 2:**
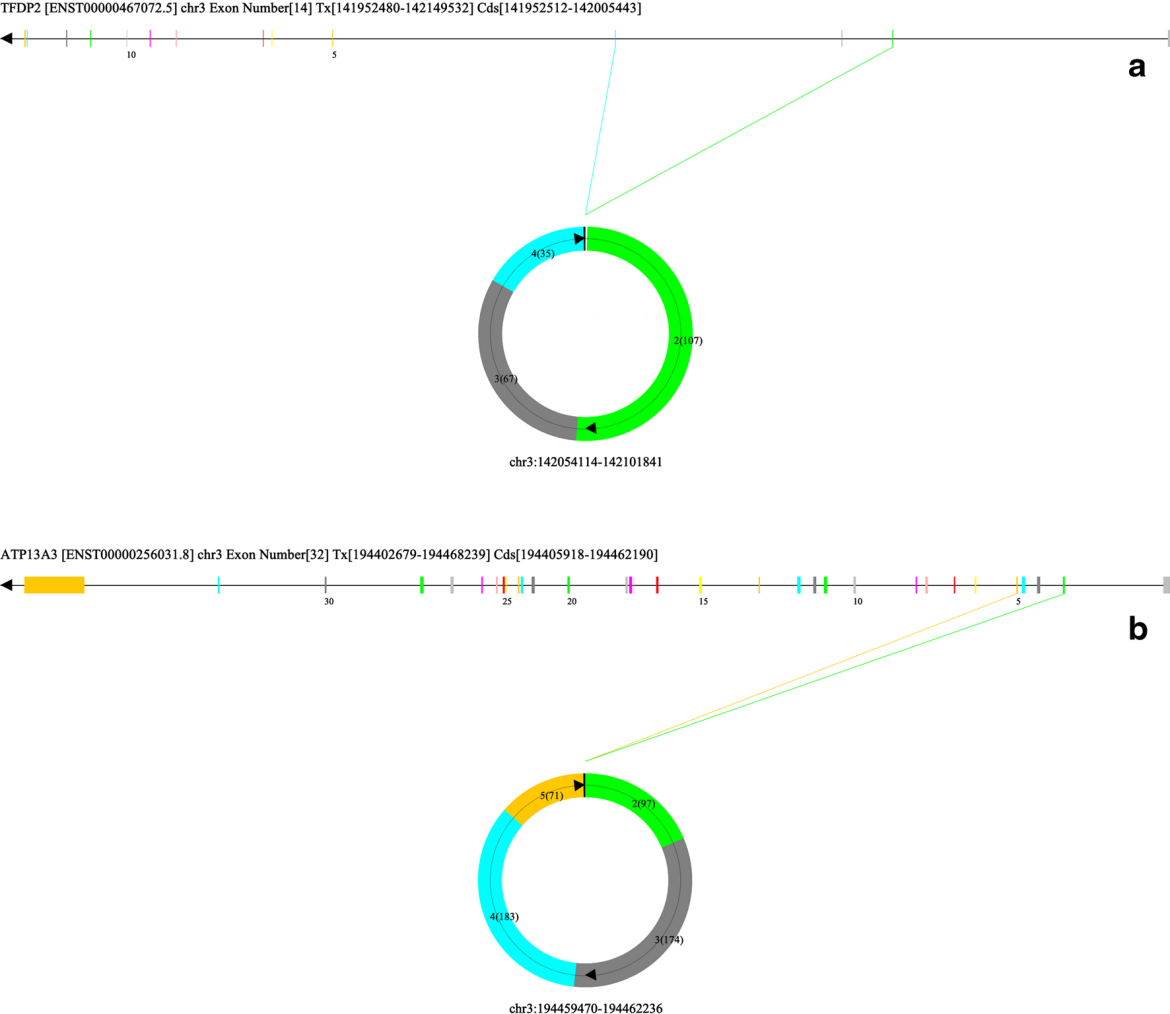
Visualisation of differentially expressed circTFDP2 and circATP13A3. The top two upregulated circRNAs listed in Table 1 are shown; the remaining eight circRNAs can be seen in Fig. S2. Gene transcript information was shown on top of each graph: (**a**) gene name. (**b**) Gene transcript ID. (**c**) Chromosome (chr) number of the circRNA. (**d**) The number of exons consisted of the gene. (**e**) Coding sequence (Cds) nucleotides coordinates. Exons are shown as coloured bars and labelled with increments of five. Introns are shown as black lines. The arrow on the gene locus indicates the direction of the transcript to be from 5′- to 3′- end. Coloured arrows correspond to the colour of exons, indicating the position of the exons within the gene locus. On the circular graph, the black bar represents the position of BSJ (5′-donor site and 3′-acceptor site), and the curved arrows represent the direction of exon involvement. On each coloured exon, the sequential exon number from the gene locus is shown and the exon lengths are displayed in brackets

Individual circRNAs were visualised using CircView, with detailed information about the expressed gene locus (Fig. 2). Exons are shown as coloured bars and labelled with increments of five. Introns are shown as black lines. The arrow on the gene locus indicates the direction of the transcript to be from 5′- to 3′- end. Coloured arrows correspond to the colour of exons, indicating the position of the exons within the gene locus. On the circular graph, the black bar represents the position of BSJ (5′-donor site and 3′-acceptor site), and the curved arrows represent the direction of exon involvement. On each coloured exon, the sequential exon number from the gene locus is shown and the exon lengths are displayed in brackets.

### Gene Ontology Terms Enrichment Analysis

**Fig. 3:**
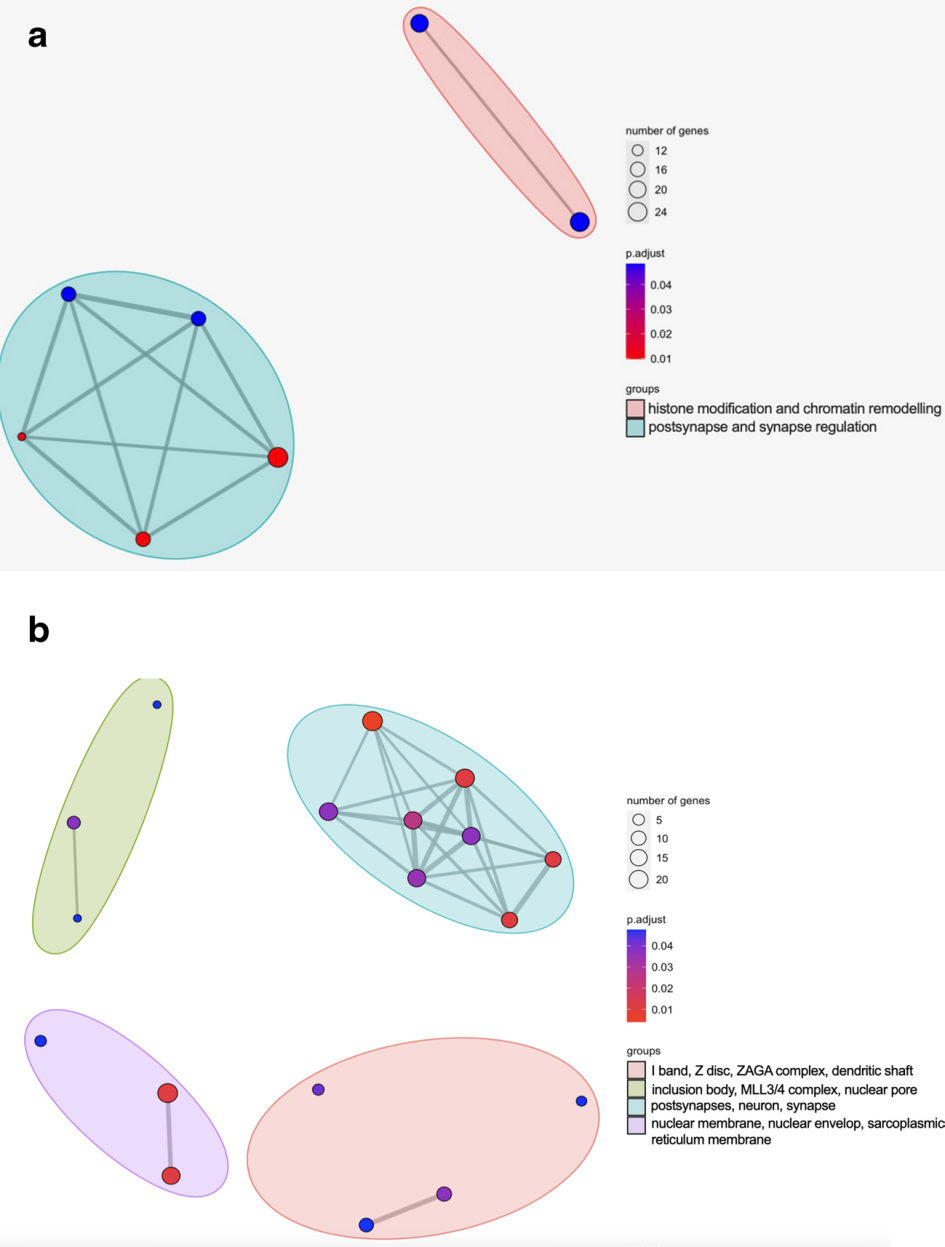
Gene ontology enrichment map for the selected circRNAs matched between CIRI2, CIRCexplorer2 and CircView outputs. **A** Biological process. **B** Cellular component. The enriched terms in the enrichment map were organised into networks. The nodes represent enriched GO terms and the node colour represents the significance of the adjust *p*-value. Similar terms were grouped into clusters. Groups were named using the abbreviation of terms names. The size of the node denotes the number of genes attributing to the enrichment

As a part of this study, a more comprehensive understanding of the implications of circRNA expression on biological processes and cellular components was completed using clusterProfiler (Fig. 3 and Table 2). Among the 120 upregulated differentially expressed circRNAs, no significant enrichment of Gene Ontology (GO) terms emerged with the cut-off *p*-value of 0.05.

**Table 2:** List of enriched GO terms for genes expressing downregulated circRNAs in AD

ID	Description	p.adjust	Count^1^
Biological process			
GO:0099173	Post-synapse organisation	0.010	16
GO:0050808	Synapse organisation	0.010	27
GO:0099175	Regulation of post-synapse organisation	0.012	11
GO:0016570	Histone modification	0.048	25
GO:0050807	Regulation of synapse organisation	0.048	16
GO:0050803	Regulation of synapse structure or activity	0.048	16
GO:0006338	Chromatin remodelling	0.048	22
Cellular component			
GO:0098978	Glutamatergic synapse	0.004	24
GO:0098984	Neuron to neuron synapse	0.011	21
GO:0043197	Dendritic spine	0.011	13
GO:0031965	Nuclear membrane	0.011	18
GO:0044309	Neuron spine	0.011	13
GO:0005635	Nuclear envelope	0.011	24
GO:0014069	Postsynaptic density	0.025	18
GO:0032279	Asymmetric synapse	0.035	18
GO:0097060	Synaptic membrane	0.037	19
GO:0099572	Postsynaptic specialization	0.037	18
GO:0031674	I band	0.037	10
GO:0016234	Inclusion body	0.038	7
GO:0043198	Dendritic shaft	0.042	5
GO:0000124	SAGA complex	0.048	4
GO:0030018	Z disc	0.048	9
GO:0044615	Nuclear pore nuclear basket	0.048	3
GO:0044666	MLL3/4 complex	0.048	3
GO:0033017	Sarcoplasmic reticulum membrane	0.048	5

^1^Count, number of genes

In contrast, to achieve higher specificity of GO enrichment, the top 500 genes expressing downregulated circRNAs were analysed, with the result demonstrating an association with seven annotated biological processes, grouped into two distinct clusters (Fig. 3 and Table 2). The enriched genes expressing the down-regulated circRNAs were involved in several biological pathways represented in a network configuration (Fig. 3A). From Fig. 3B, the cellular component analysis for the downregulated circRNAs revealed 18 annotated terms grouped into four clusters. This suggests the downregulated circRNAs in AD brain might play a role in influencing the structural organisation of cells.

There was no significant GO terms enrichment for the selected top 500 downregulated linear transcripts. However, genes expressing upregulated linear RNAs were enriched and linked significantly to one biological process: “one-carbon compound transport”.

### KEGG Pathway Analysis

**Fig. 4:**
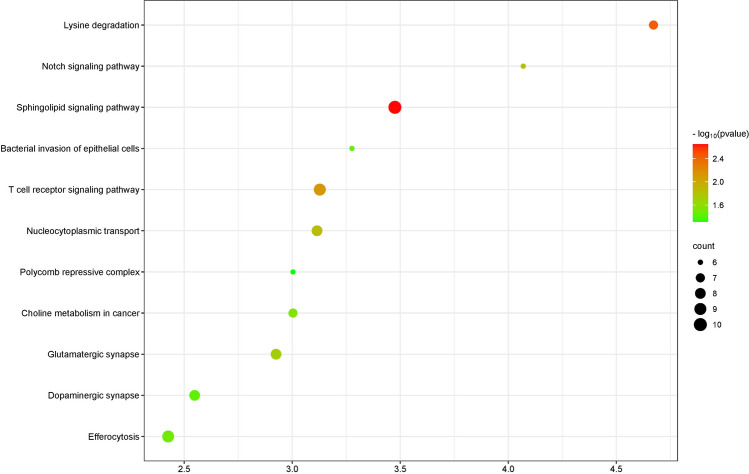
KEGG pathway analysis of down-regulated circRNAs (*p*-value < 0.05). *y*-axis represents the enriched KEGG pathway. The size of the nodes represents the number of genes involved for each enrichment. The colour of the nodes represents the significance of the *p*-value

To further analyse and interpretate the functional meaning of the dysregulated circRNAs, genes of the significantly differentially expressed circRNAs were subjected to KEGG analysis. Among the top 500 downregulated circRNAs, sphingolipid signalling pathway, lysine degradation and T-cell receptor signalling pathways were most related to the target genes (*p*-value < 0.01) (Fig. 4). KEGG pathway analysis revealed that only one pathway named as Apelin signalling pathway (*p*-value < 0.05) was associated with genes expressing the top 120 upregulated circRNAs.

### circRNA-miRNA-mRNA Network

The CRAFT tool interlinks with multiple types of databases (miRbase, GeneCards, NCBI Entrez, Ensembl, Uniprot and NCBI Pubmed) in to order to generate functional prediction result. It delivers several predicted miRNA-binding sites and miRNAs for the input circRNA IDs and sequences (Table 3). From the ten selected upregulated circRNAs (Table 1), four circRNAs were identified to express miRNA-binding sites.

**Table 3:** Four circRNAs, upregulated in AD, display enrichment of miRNA-binding sites

CircRNA	Gene name	Number of miRNA-binding sites	Number of miRNAs	CircRNA length
1:10149,184–10161286	UBE4B	549	263	12,103
X:14843,660–14865580	FANCB	17	16	2638
11:86012281–86031611	PICALM	7	7	654
2:169538013–169544835	FASTKD1	4	4	373

The CRAFT software predicted a number of miRNAs-binding sites for each of the ten circRNAs (Table S4). A previous study assumed that the number of specific miRNA-binding sites is greater than seven (Hall IF et al*.*, 2019). Hence, of those which are selected for the miRNA sponges’ prediction, circUBE4B contained the highest number of miRNA-binding sites, and it was the only circRNA acting as potential miRNA sponges (Table 4). Among the list of predicted miRNAs, hsa-miR-4739 comprised of the highest frequency of miRNA-binding sites and highest density value of 0.1.

**Table 4:** miRNA-sponging characteristics of circUBE4B in the chromosomal location chr1:10,149,184–10161286

miRNA	Number of miRNA-binding sites	Density^1^
hsa-miR-4739	12	0.1
hsa-miR-3192-5p	11	0.09
**hsa-miR-328-5p**	**10**	**0.08**
hsa-miR-6778-5p	10	0.08
hsa-miR-6848-5p	10	0.08
hsa-miR-5787	9	0.07
hsa-miR-6771-5p	9	0.07
hsa-miR-1233-5p	8	0.07
hsa-miR-1249-5p	8	0.07
hsa-miR-4695-5p	7	0.06
hsa-miR-4728-5p	7	0.06
hsa-miR-6754-5p	7	0.06
hsa-miR-7851-3p	7	0.06

^1^Density = ratio of number miRNA-binding sites to circRNA length; miRNA shown in bold has *CD44* mRNA as one of its targets (see Fig. 6 for details).

**Fig. 5:**
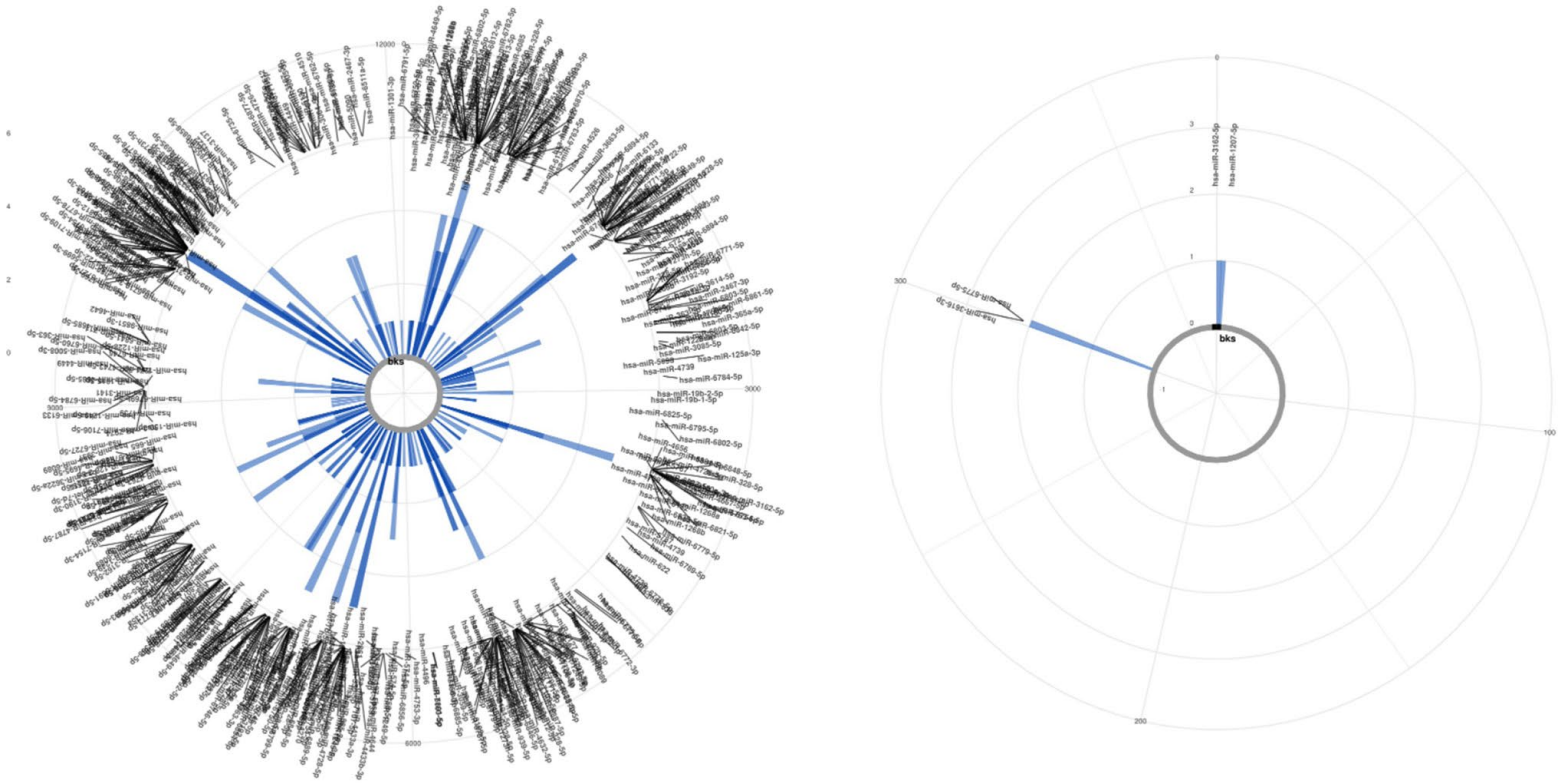
Graphical visualisation of miRNA-binding sites along sequences of circUBE4B (**A**) and circFASTKD1 (**B**). The small grey circle at the centre represents the respective circRNA. Each extended blue bar represents predicted miRNAs. The sequential numbers positioned vertically at the middle of the circle represent the number of miRNAs sharing identical MRE start position. The outermost layer of the circle, delineated by sequential numbers in a clockwise direction represents the circRNA sequence length

Circular plots on miRNA section generated for circUBE4B and circFASTKD1 (Fig. 5) using CRAFT software were selected for comparison of visualisation. Each plot displays all predicted miRNAs and their respective MREs for the designated circRNA. The name of the predicted miRNAs’ and their MREs’ positions on the specific circRNAs were retrieved from miRBase, which is a miRNA database integrated within the CRAFT software. Circular plots for circFANCB and circPICALM are in Fig. S3.

**Fig. 6:**
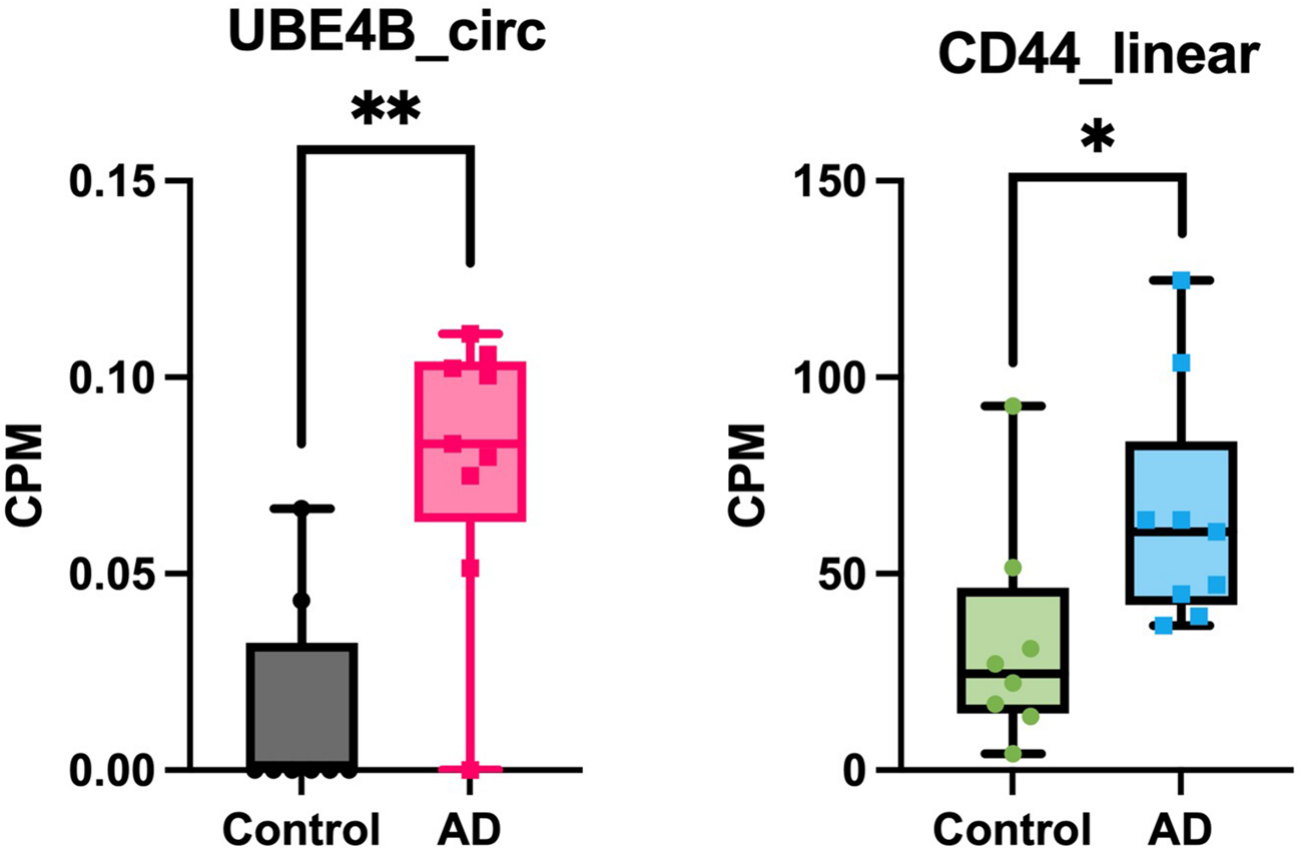
CircUBE4B and *CD44* mRNA expression in AD and control samples. The number of asterisks represent the level of significance of the CPM value in AD comparing to control samples (**p-value < 0.01, *p-value < 0.5). For circUBE4B, the control and AD samples were presented in grey and pink colours, respectively. For the *CD44* mRNA, the control and AD samples were presented in green and blue colours, respectively

To investigate the circRNA-associated competing endogenous RNA (ceRNA) regulatory network, CRAFT tool revealed that circUBE4B, through predicting mRNA target associated with hsa-miR-328-5p (Table 4), potentially regulates the expression of the *CD44* gene (Fig. 6 and Table S3). None of the other target genes expressing mRNAs predicted by CRAFT tool for miRNAs listed in Table 4 were significantly differentially expressed in this analysis.

## Discussion

CircRNAs are a class of non-coding RNAs that have yet to be extensively explored in the context of neurodegenerative diseases. According to the pathogenesis of neurodegenerative disease, neuronal loss directly leads to less proficient neuronal maintenance and synaptic transmission (Tom SE et al. 2015). This study aims to deepen the understanding of the circRNA transcriptome within the Alzheimer’s disease (AD) landscape and subsequently expand on the initial discovery of circRNA expression in AD. By conducting a differential expression analysis of circRNAs in nine AD samples and eight control samples followed by the visualisation of selected circRNAs, and investigation on the circRNAs functions.

### Transcriptome Sequencing Metrics

#### Lower Measurement of circRNAs Recovered than Linear RNAs

An intriguing finding centres around the distribution of low counts per million (CPM) values (CPM < 1) among the top ten selected circRNAs in both AD and control samples (Table 1). In contrast, their linear counterparts showcase notably higher CPM values. This pattern of lower circRNA abundance aligns with prior findings and echoes established trends in circRNA expression levels (Aquilina-Reid C et al. 2022; Cheng Y et al. 2023; Takenaka K et al. 2023). A study by Guo et al. (Guo JU et al. 2014) suggested that low abundance of circRNAs in mammalian tissues was a result of imperfect pre-mRNA splicing. Back-splicing of circRNAs is regulated by multiple elements, which modulates the back-splicing event to be less efficient.

It is worth noting that the identification of circRNAs is restricted to the detection of back-splice junction (BSJ) sequences within aligned reads (Feng J et al. 2017; Gao Y et al. 2015; Zhang XO et al. 2016), while linear RNAs are identified based on the short reads that match the reference gene loci (Pertea M et al. 2015). CircRNA detection algorithms aim to produce comparatively reliable results through minimising the false positive rates, which involves the use of gene annotations or canonical splice signals. However, these strategies were found to impact the algorithms sensitivity (Szabo L and Salzman J, 2016). To increase the accuracy of this study, common circRNAs detected by CIRI2, CIRCexplorer2 and CircView were filtered as considering the algorithm-specific criteria for BSJ-aligned reads.

#### A Higher Number of Downregulate circRNAs in AD Samples

**Fig. 7:**
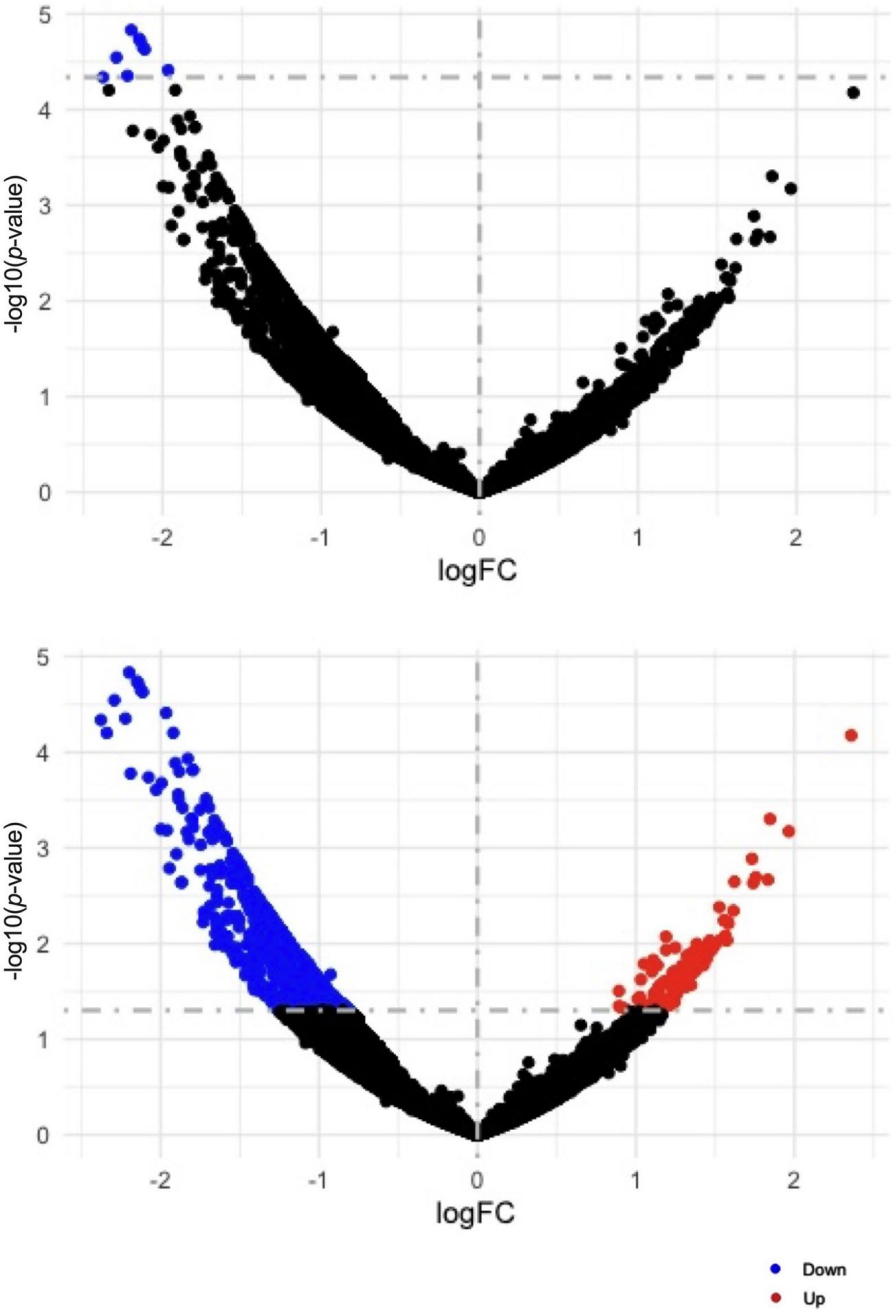
Volcano plots of differentially expressed circRNAs in AD compared to control subjects. Differentially expressed circRNAs are based on an adjusted *p*-value < 0.1 (**A**), and unadjusted *p*-value < 0.05 (**B**). Downregulated circRNAs are presented in blue and upregulated circRNAs are presented in red. The plot was generated with − log_10_ of the *p*-values and fold change (FC) on the axis

A differential expression analysis for circRNAs and their linear counterparts (Fig. 7) in between AD samples and control samples was completed. In this study, a higher number of downregulated circRNAs and genes were identified, which is reflective in other neurodegenerative diseases such as Parkinson’s disease and Huntington’s disease (Kong F et al. 2021; Li MD et al. 2014). A higher number of downregulation of circRNAs might indicate the disruption of relevant interactions and downstream pathways such as gene regulatory abilities as well as interactions with miRNAs and RNA-binding proteins (Ashwal-Fluss R et al. 2014; Goodrich JA and Kugel JF 2006; Hansen TB et al. 2013; Pamudurti NR et al. 2017). For instance, dysregulated expression of circRNAs in the brain impacts multiple downstream responses. Previous studies AD have demonstrated circRNAs in the brain which are involved in Aβ generation and clearance, neuroinflammation, neuronal oxidative stress and autophagy in (González-Scarano F and Baltuch G, 1999; Huang J-L et al. 2020; Lukiw WJ et al. 2015; Wang H et al. 2019; Zhou Z-b et al. 2018). Therefore, the discovery of dysregulation of AD-associated circRNAs indicates neuronal and synaptic dysfunction in AD brains.

### Significance of the Differentially Expressed circRNAs

#### Uniquely Differentially Expressed circRNAs

This study revealed uniquely differentially expressed circRNAs that have not yet been mentioned in AD including circATP13A3, circFANCB and circFASTKD1 (Table 1). As these uniquely circRNAs are only observed in the AD samples, it implies the potential unknown pathways or interactions of the molecules that are involved in circRNAs regulations and potentially AD-specific pathogenic mechanisms. Notably, this pattern of dysregulation is not mirrored in the linear counterparts of these circRNAs, which exhibit no significantly differential expression between AD and control samples (Fig) (Rybak-Wolf A et al. 2015). A study using poly(A) + RNA samples obtained from AD brains also displayed no correlation in expression levels between the most abundant circRNAs and their mRNA counterparts through ranking (Arizaca Maquera KA et al. 2023). The heightened expression of dysregulated circRNAs could potentially serve as indicative markers of their involvement in AD pathology.

#### Selected Upregulated circRNAs in This Study and Their Associated Linear Products Found in Previous Studies

Among the group of top ten upregulated circRNAs (Table 1), none have previously been identified as differentially expressed in AD brains. However, their linear RNA counterparts or the resultant protein products have been linked to AD pathogenesis. For instance, the upregulated Septin7 protein has been implicated in molecular reactions within AD mice, implying its potential significance in disease processes of AD in humans (Wang X et al. 2018). Similarly, the ubiquitin conjugation E4B (UBE4B) protein’s ability to target the miR-9 gene contributes to tau tangle degradation in AD patients, further reinforcing its role in disease modulation (Subramanian M et al. 2021). Previous studies have demonstrated implications of downregulated circFASTKD1 and circGF2I (Gao WQ et al. 2020; Yuan C et al. 2022) associated with myocardial infarction. Similarly, these circRNAs have also been observed in an intersection between cardiovascular and neurodegenerative disease pathways (Huang L-Y et al. 2023). Furthermore, the linear counterpart of the *PICALM’s* dysregulation influences the risk of AD development by impacting APP processing, Aβ transcytosis and tau progression, highlighting its multifaceted influence on AD pathogenesis (Ando K et al. 2022).

#### Statistically Significantly Upregulated circRNAs in AD and Relevant Studies

Amongst the 120 statistically significantly upregulated circRNAs (Table S2), three were previously described as being upregulated in AD in literature (Li Y et al. 2020; Ma N et al. 2020; Song C et al. 2022). For instance, the upregulation of circPCCA was validated through microarray analysis (*p*-value < 0.001) and exhibited increased expression in AD, hinting its potential as a robust marker for the diagnosis of AD (Li Y et al. 2020). Similarly, circCwc27, which is abundantly expressed and significantly upregulated in AD, may be promising as a therapeutic target for cognitive dysfunction in AD patients (Song C et al. 2022). Another example is circPTK2, which warrants further investigation into its potential involvement in AD processes (Ma N et al. 2020). The identification of these circRNAs in AD samples validates the analytical pipeline and findings in this project and provides an understanding to the other upregulated circRNAs in AD pathology.

#### Dysregulation of circRNAs Comparing to Their Linear Host Transcripts

In this study, the circRNA abundance and levels of dysregulation are independent to their linear transcripts (Fig. 1). It is known that back-splicing requires spliceosome and canonical splice sites to produce circRNAs (Ashwal-Fluss R et al. 2014). A recent study (Liang D et al. 2017) has revealed that the depletion of components of the U2 small nuclear ribonucleoprotein (snRNP) spliceosome could promote higher expression of circRNAs whilst reducing mRNA production. Liang et al. (Liang D et al. 2017) proposed that canonical splicing necessitates the transition promoted by U1/U2 snRNP from cross-exon interaction to cross-intron interaction, compared to canonical back-splicing. On the other hand, the U4/U5/U6-tri-snRNP components’ spliceosome directly promotes circularisation (Schneider M et al. 2010; Starke S et al. 2015). Hence, inhibition of some subunits of spliceosome might improve circRNAs expression, while not significantly affect levels of canonical splicing. This supports the findings in this study where only competent circRNAs were statistically significantly upregulated in this project, but their linear host mRNAs exhibit no significantly differential expression.

### Gene Ontology Enrichment and KEGG Pathway Analysis for Differentially Expressed circRNAs

The preliminary GO enrichment analysis for downregulated circRNAs included numerous terms related to the nervous system including synapse, neurons and dendrites, which are related to the affected AD tissues (Griffiths J and Grant SGN 2023) (Table 2; Fig. 3). Disruptions of synapse formation and maintenance directly contribute to cognitive decline, a characteristic symptom of AD (Dorostkar MM et al. 2015). Among the biological process GO terms, processes related to synapse organisation and activity were prominent, constituting five out of the seven processes. This implicates strong correlation between the downregulation of the host genes expressing these circRNA and the symptoms of AD. Although the GO analysis is based on protein products derived from linear RNAs, the association between circRNAs and linear transcripts should not be ignored. It has been noted that circRNAs are able to influence the expression of linear transcripts by competing for mRNA splicing machinery (Ashwal-Fluss R et al. 2014). It insinuates a possibility for them to indirectly contribute to the onset of AD symptoms through this mechanism. Further evidence of downregulated circRNAs contributing to the pathogenesis of AD is observed from the cell component GO terms results, with 11 out of 18 terms related to the nervous system (Fig. 3A). The most significant cell component term was glutamatergic synapse, where its disruption has been observed to be one of the most important pathological indicators of cognitive decline for AD (Coleman PD and Yao PJ 2003). The dysregulation of one-carbon compound transport has been reported to result in increased homocysteine levels in circulation, which was accompanied by AD progression and cognitive decline (Smith AD and Refsum H, 2016). Other enriched terms such as neuron to neuron synapse, dendritic spine and postsynaptic density have been reported and are consistent with the current literature on AD progression (Dorostkar MM et al. 2015; Griffiths J and Grant SGN 2023; Vyas Y and Montgomery JM 2016). The corroboration from other studies reinforces the notion that downregulated circRNAs contribute to AD’s pathophysiological development and progression.

According to the Kyoto Encyclopedia of Genes and Genomes (KEGG) enrichment, the downregulated circRNAs enriched several signalling pathways (Fig. 4) which have been reported previously to be significant for AD pathogenesis. Sphingolipids, a highly enriched pathway in this KEGG analysis, were able to contribute to the biogenesis regulation of Aβ, tau, ⍺-Syn, and APOE that are biomarkers of AD pathogenesis (Wang X et al. 2024). For example, sphingolipids interact with Aβ oligomers to form endogenous GAβ seeds which urge the aggregation of extracellular Aβ plaques, leading to cell apoptosis and neurotoxicity in AD brain (Matsuzaki K, 2020). Decrease of lysine metabolism was observed in hippocampus region of AD brain tissue (Xu J et al. 2016). CircPSEN1 is involved in lysine degradation in autosomal-dominant AD patients (Chen HH et al. 2022). Evidence indicates that the dysfunction of T-cell receptor signalling pathway in immunological and pathological regulation could potentially modulates the cell homeostasis and neuroinflammatory response in AD (Browne TC et al. 2013; Dai L and Shen Y, 2021). Genes expressing the downregulated circRNAs are enriched in nucleocytoplasmic transport, which are responsible for the damage of the nuclear pore complex or other impairment of factors in AD (Nag N and Tripathi T, 2023). Notch signalling pathway is a conserved cell signalling control involved in vascular and cerebrovascular development and function (Knopman DS et al. 2003). Mutation of the *PSEN1* identified in this study was demonstrated to induce Aβ and neuronal activity at the stage of human-induced pluripotent stem cell-derived cortical spheroids modification. It was also found to increase Notch signalling in familial AD (Hurley EM et al. 2023). More than 40% of neuronal synapses are glutamatergic synapses; therefore, the accumulation of neurotoxicity is recognised as a result of the abnormality of glutamatergic synapses and dysregulation of glutamate (Bukke VN et al. 2020; Cassano T et al. 2012). Efferocytosis in AD brain tissue denotes for the clearance of apoptotic neurons and Aβ by phagocytosis (Tajbakhsh A et al. 2021), which brings over a positive effect for AD brain. The “bacterial invasion of epithelial cells” from the reported KEGG pathway can be explained when AD-related pathological activity contributes to and the entry of the noxious microbiomes into the circulation through the leaky blood–brain barrier dysbiosis in AD patients (Bulgart HR et al. 2020). Moreoever, reducing dopaminergic neurons correlates with memory impairment and reward dysfunction at pre-plaque stage (Nobili A et al. 2017). Polycomb repressive complex from the KEGG pathway was known to prevent and downregulate the expression of harmful genes via methylation process in AD (Cholewa-Waclaw J et al. 2016; Kouznetsova VL et al. 2019). By implementing the top 120 upregulated circRNAs to KEGG pathway analysis, *RYR2I* and *PIK3C3* were reported to have involvement in the apelin signalling pathway. Upregulation of *RYR2* leads to higher probability deteriorates neuronal dysfunction in familial AD via processing modification on neuronal hyperactivity (Yao J and Chen SRW 2024). Study from Yang C et al. (2017) uncovered that the PIK3C3 protein is associated with a complex that is able to disrupt APP metabolism and Aβ homeostasis. Of note, the annotation of GO or KEGG enrichment analysis is still based on protein coding gene, which results in less interpretation on intrinsic functions of the circRNAs (Cheng Y et al. 2023).

### CircRNA-miRNA-mRNA Network

Several studies have revealed the regulatory potential for circRNAs during the circRNA-miRNA-mRNA network in development of AD (Li Y et al. 2022; Lu Y et al. 2019; Ma N et al. 2019; Zhang Q et al. 2022). The competitive endogenous network suggests the noncoding RNAs competes with other RNAs to bind to miRNAs for regulatory function (Gao L et al. 2021; Salmena L et al. 2011). The investigation of miRNA-binding sites for the selected circRNAs revealed circRNAs’ unique ability to bind to their target miRNAs (Table 3). It is predicted that the upregulated circUBE4B has the capacity to act as a potent miRNA sponge to multiple miRNAs (Table 4). Of note, the functional role of gene expression regulation for circUBE4B is manifested through its targeting of gene *CD44* via hsa-miR-328-5p, whose linear transcript from gene *CD44* was also observed to be upregulated in AD samples. This corroborates with the statement that circRNAs as a miRNA sponge leads to inhibition of miRNA from binding to its target gene, consequently leading to the expression of linear RNA of the target gene. Tan et al.’s study in 2021 illustrated the correlation of miR-328-3p with multiple target genes expressing mRNAs in AD pathogenesis. Notably, miRNA strand denoted with − 3p or − 5p suffix represent the mature miRNA derived in either 3′ – 5′ prime end direction or 5′ – 3′ prime end direction, respectively (Kozomara A and Griffiths-Jones S, 2014). Referring to the miRBase database (https://mirbase.org/), both hsa-miR-328-3p/5p strands are functional but might involve distinct regulatory potentials in neurons due to their structural difference (Kim J et al. 2004). From previous studies, *CD44* serves as a marker for microglial and astrocytic activation in AD brain (Akiyama H et al. 1993; Pesämaa I et al. 2023). It was known that *CD44* correlates with a neuroinflammatory marker called chitinase-3-like protein 1 (also known as YKL-40), which is involved in Aβ and neurofibrillary tangles development in AD pathogenesis (Craig-Schapiro R et al. 2010). Based on previous finding about roles of YKL-40 in repair and remodelling (Zhao T et al. 2020), it was hypothesised that *CD44* and YKL-40 are responsible for neuroprotection during cognitive impairment (Kognole AA and Payne CM 2017; Moreno-Rodriguez M et al. 2020; Toole BP 2009). Overall, circUBE4B targets the trafficking of hsa-miR-328-5p and promotes expression of *CD44* in brain region of dorsolateral prefrontal cortex of AD patients, thereby implicated in the pathology of AD. From a clinical perspective, circUBE4B might serve as a potential biomarker for AD diagnosis and treatment, referring to its regulatory role in the circRNA-miRNA-mRNA competitive endogenous network (Memczak S et al. 2013). This finding supports the understanding that circRNAs is pivotal for complex molecular interaction and expression regulatory.

## Concluding Remarks

As the roles of AD-specific circRNAs remain relatively unexplored, the profiles of circRNAs uncovered in this study hold the potential to serve as biomarkers for early AD diagnosis and disease progression monitoring. Furthermore, although the functions of circRNAs within biological processes are not fully elucidated, the insights derived from this study underscore their potential significance, warranting further investigation.

Notably, one of the limitations of this study is the relatively small AD sample size (Scheckel C et al. 2016). The small sample size potentially contributes to no differentially expressed linear transcripts with adjusted *p*-value < 0.1 (Fig. S1). The tool used for Gene Ontology enrichment analysis requires enriched genes and gene clusters to calculate and generate enriched pathways (Yu G et al. 2012). This implies that less genes from a smaller sample size might not show relevant pathways due to the lack of enriched genes. The lack of GO enrichment in this study for the upregulated circRNAs indicated a controversial statement in terms of circRNAs function in AD and Gene Ontology annotation. A previous study by Tomczak et al. (Tomczak A et al. 2018) on the evolution of GO and its annotation suggested a continuous upgrade of GO annotations might deliver different interpretation of the 120 upregulated circRNAs and AD pathology. Additionally, the annotation of GO terms is still based on the discovered function of the proteins encoded by genes, which results in insufficient interpretation of the intrinsic functions of the circRNAs (Cheng Y et al. 2023). Hence, larger sample sizes and diverse brain regions could provide a more comprehensive picture of circRNA dysregulation in AD.

Regarding the circRNA detection tools used in this study, there is a sense of ambiguity in detecting the BSJs for circRNA identification as other biological molecules also naturally exhibit BSJs (Jeck WR and Sharpless NE 2014). From the evaluation of circRNAs detection methods on the RNA-seq datasets (Nguyen MH et al. 2022), CIRI2 and CIRCexplorer2 both revealed a comparatively satisfactory performance on circRNAs detection. It has been reported that the true discovery rates for these two tools remain below 80%, which is an acceptable value due to their aim of detecting as many circRNAs as possible (Nguyen MH et al. 2022). In this project, to minimize the number of false positives, common circRNAs detected by both CIRI2 and CIRCexplorer2 were merged and demonstrated that some AD-associated circRNAs detected by single algorithm were discarded from downstream analysis. Consequently, the potential loss of these circRNAs might compromise the accuracy, sensitivity and reliability of the subsequent results. The advent of long-read sequencing such as Oxford nanopore technology, which allows construction of full length circRNA sequence, eventually facilitates large-scale detection of circRNAs independent of BSJs detection (Rahimi K et al. 2021).

As the author (Dal Molin A et al*.*, 2022) mentioned in their paper about CRAFT assessment and parameters optimisation, the sensitivity of this tool combining both miRanda and PITA databases for MREs detection remained ranging from 0.36 to 0.71 depending on the threshold setup. Although the integration of experimentally determined AGO2-binding sites might improve sensitivity for CRAFT, the number of predictions declined to 6%. Hence, CRAFT tool was built with low stringency in order to generate the targets for circRNA-miRNA-mRNA as complete as possible. In this project, the input parameters were set as default for prediction, which compromises the accurate filtering and interactions between the ceRNAs.

Additionally, exploring the potential of circRNAs as therapeutic targets or diagnostic biomarkers requires validation in independent cohorts and longitudinal studies. The advent of long-read sequencing like nanopore, which allows construction of full-length circRNA sequence, eventually facilitates large-scale detection of circRNAs independent of BSJs detection. In prediction of circRNAs-miRNAs-mRNAs targets and interactions, Circr (Dori M et al. 2022), a newly developed computational tool that also works on detecting these interactions can be used for validation. To validate the predicted circUBE4B-hsa-miR-328-5p-CD44 network, laboratory experiment is required. For example, RNA immunoprecipitation and fluorescent in situ hybridisation can be used to identify visualise circRNA-miRNA bindings and localisation. Additionally, reverse transcription quantitative polymerase chain reaction (RT-qPCR) and dual luciferase assay can help verify the targeting relationship in context of expression between the miRNA with the target gene (Li Y et al. 2022; Luo Q et al. 2023).

Despite these constraints, the identification of differentially expressed circRNAs and circRNA-miRNA-mRNA network within the dorsolateral prefrontal cortex of AD brains presents new avenues for future experimental exploration. In conclusion, the complex landscape of circRNAs in the context of neurodegenerative diseases, particularly Alzheimer’s disease, remains an area of great complexity including the pathogenesis and interactive pathways. As our understanding of the roles of circRNAs deepens, it opens doors to novel diagnostic and potential therapeutic avenues for neurodegenerative diseases that urgently need effective interventions.

## Supplementary Information

Below is the link to the electronic supplementary material.

Supplementary file1 (JPEG 77 KB)

Supplementary file2 (PDF 19 KB)

Supplementary file3 (PDF 522 KB)

Supplementary file4 (JPEG 308 KB)

Supplementary file5 (JPEG 302 KB)

Supplementary file6 (JPEG 185 KB)

Supplementary file7 (PDF 365 KB)

Supplementary file8 (JPEG 269 KB)

Supplementary file9 (PDF 3313 KB)

Supplementary file10 (XLSX 17437 KB)

Supplementary file11 (XLSX 1539 KB)

Supplementary file12 (XLSX 6512 KB)

Supplementary file13 (XLSX 17 KB)

## Data Availability

Data is provided within the manuscript or supplementary information files.
