# Supplementary figures and images for: Investigation of the Circular Transcriptome in Alzheimer’s Disease Brain
Source: J Mol Neurosci. 2024 Jul 9;74(3):64. doi: 10.1007/s12031-024-02236-0 (PMC11233389; doi:10.1007/s12031-024-02236-0)

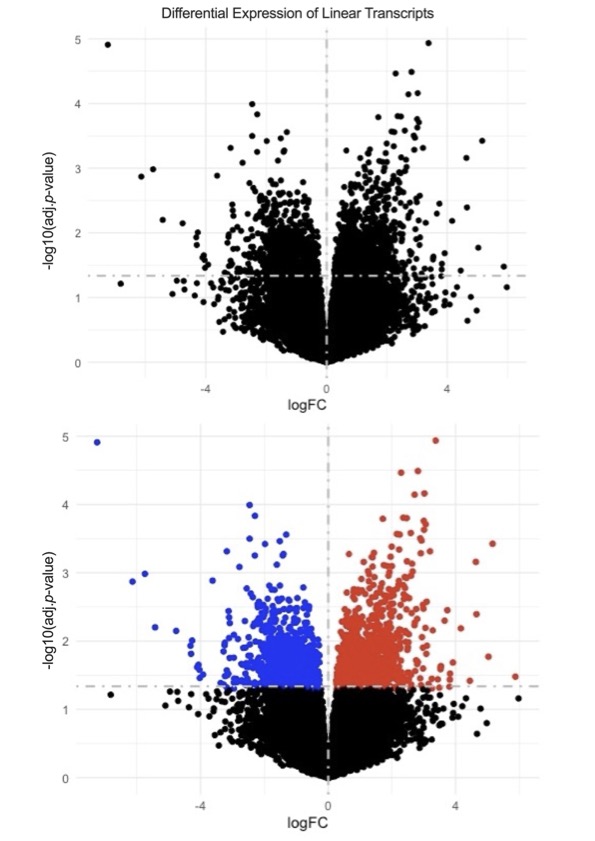

Supplement: Supplementary file 1 — Supplementary file1 (JPEG 77 KB) [file 12031_2024_2236_MOESM1_ESM.jpeg]

Differential Expression of Linear Transcripts

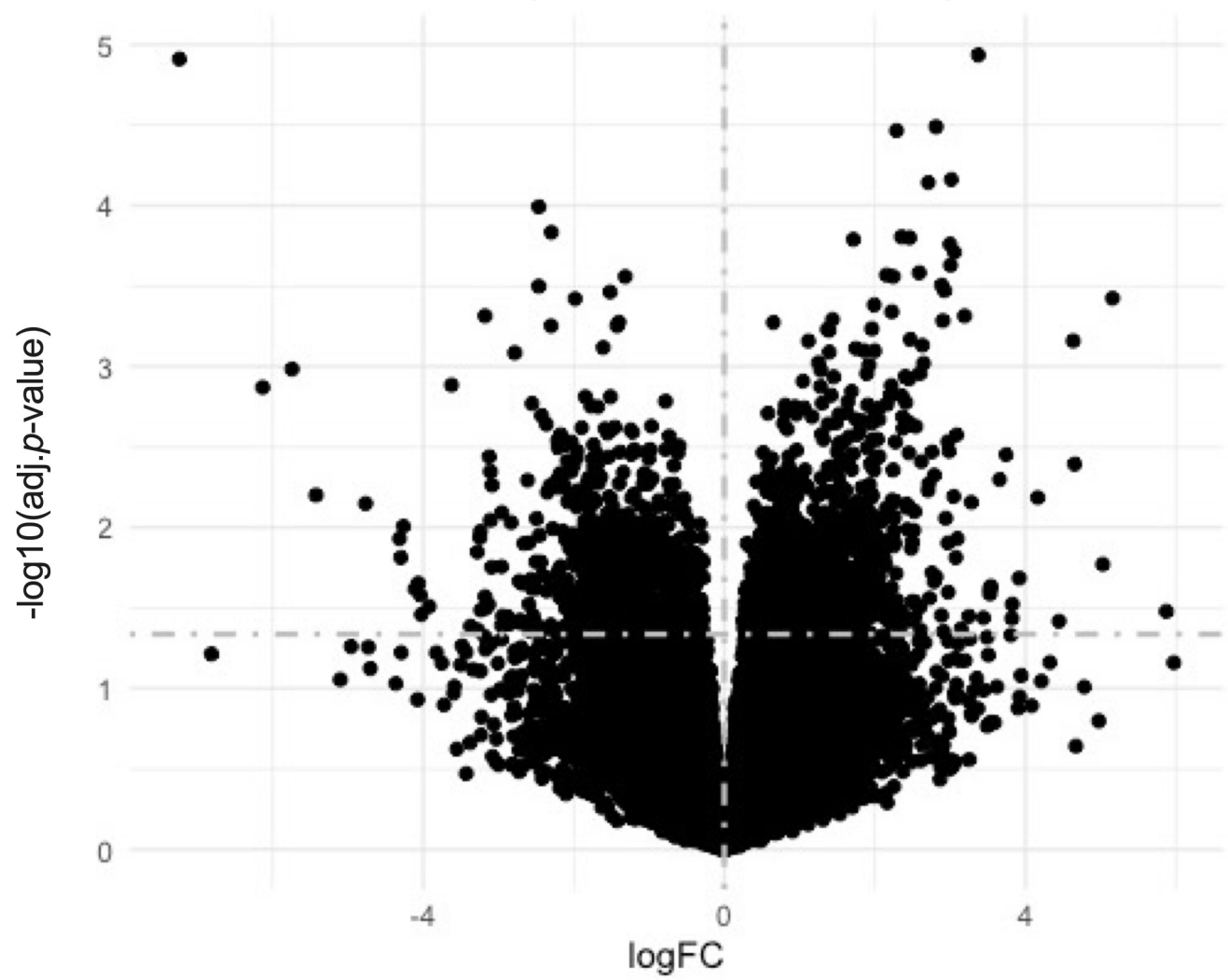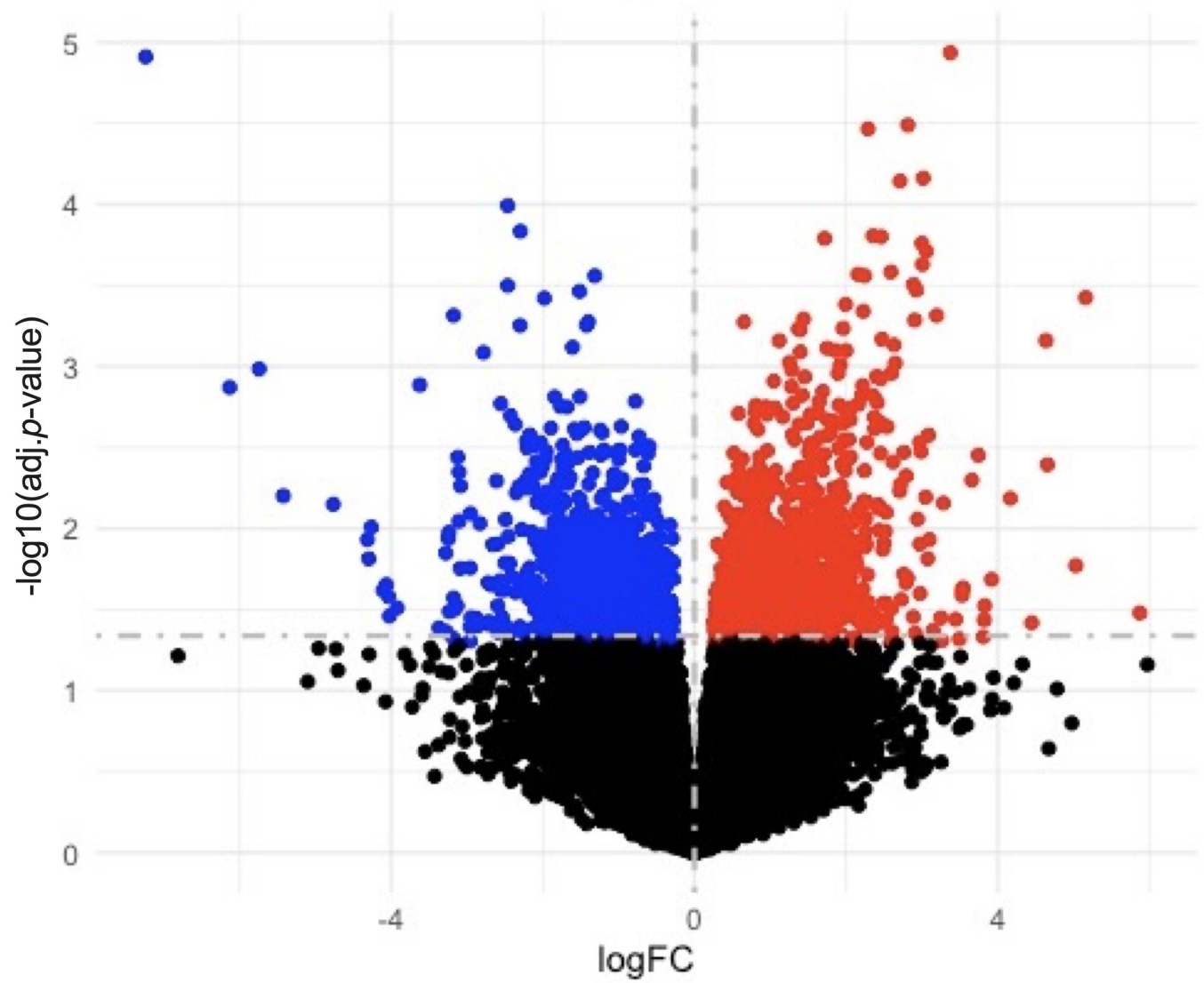

Supplement: Supplementary file 3 — Supplementary file3 (PDF 522 KB) [file 12031_2024_2236_MOESM3_ESM.pdf]

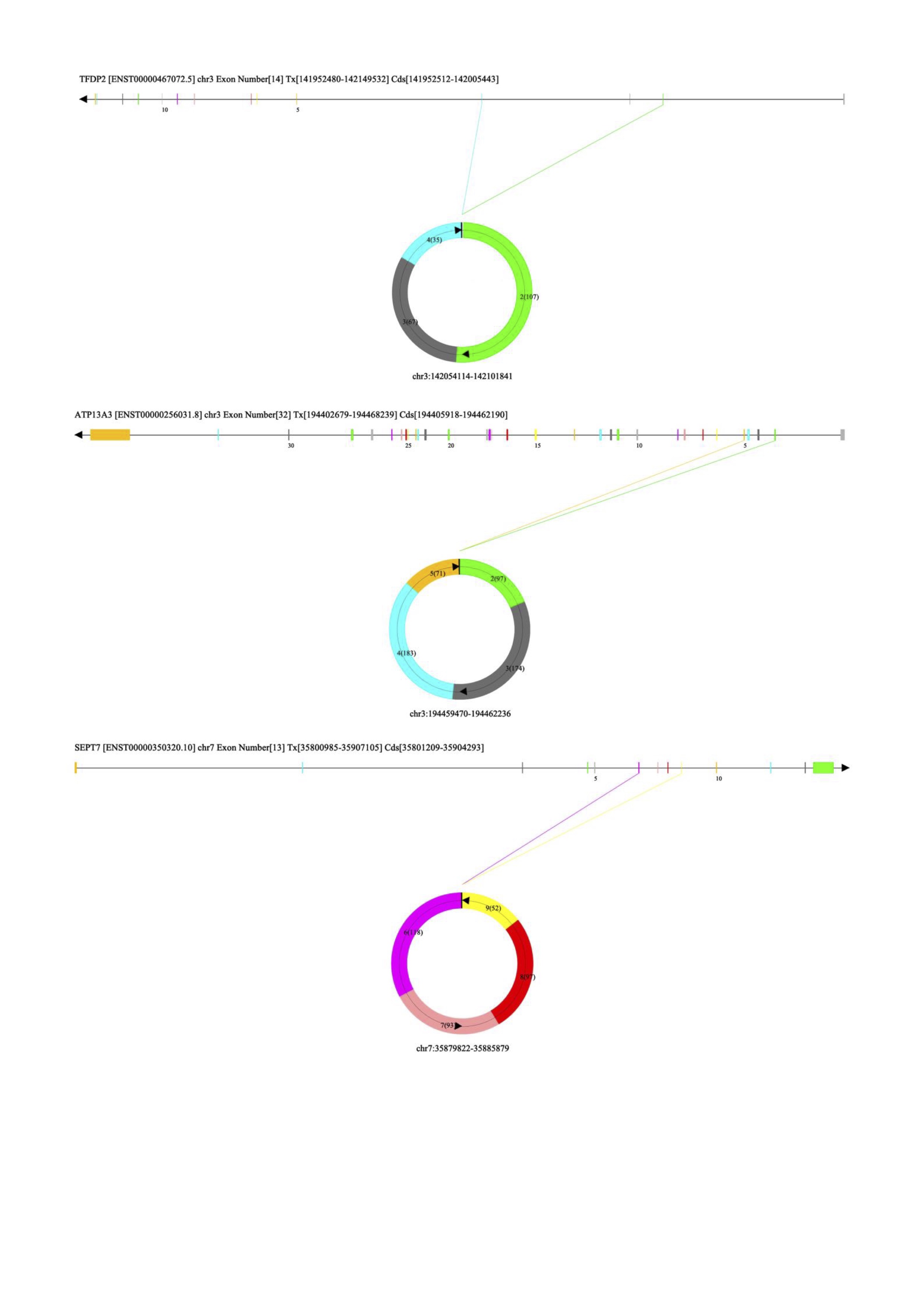

Supplement: Supplementary file 4 — Supplementary file4 (JPEG 308 KB) [file 12031_2024_2236_MOESM4_ESM.jpeg]

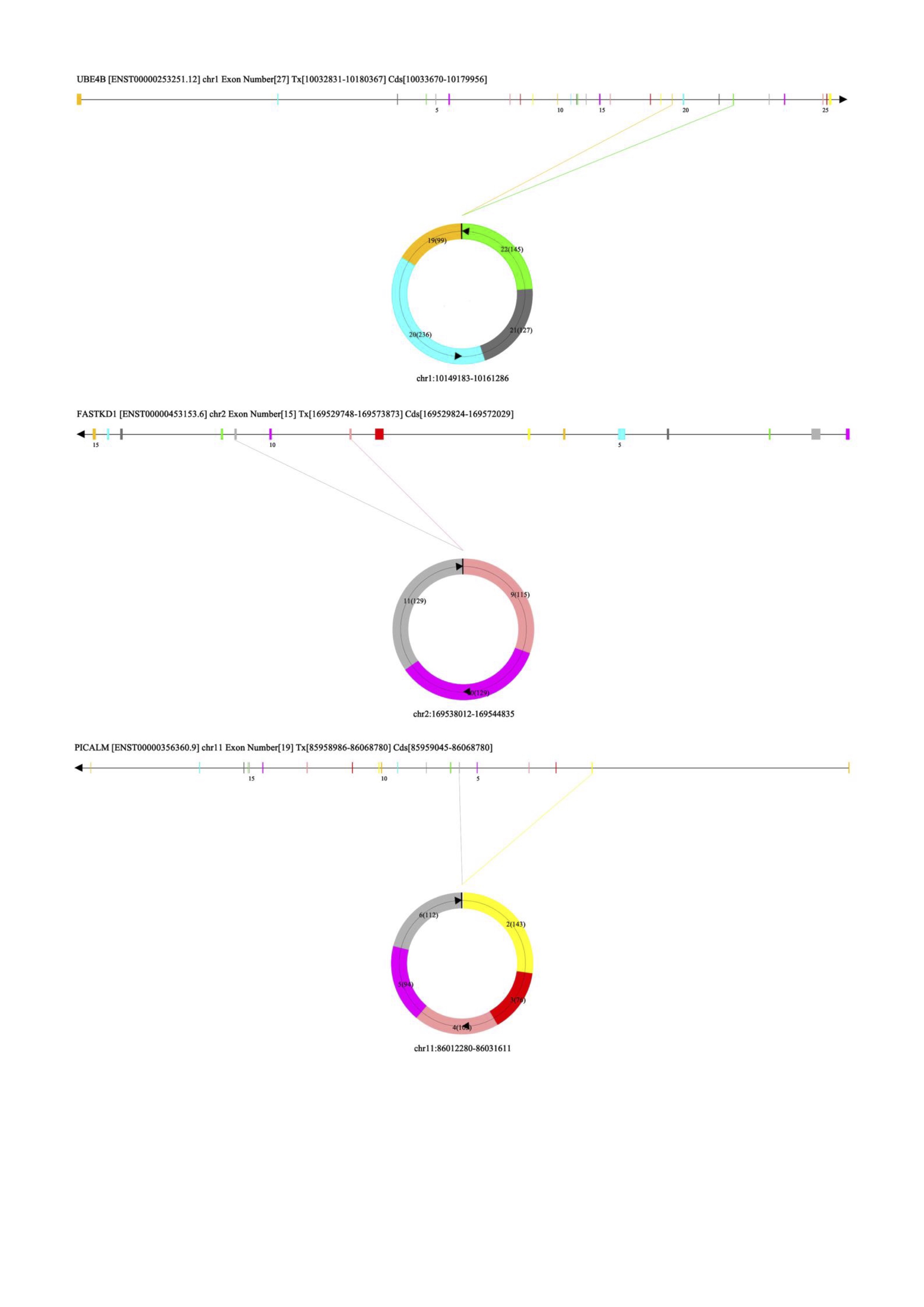

Supplement: Supplementary file 5 — Supplementary file5 (JPEG 302 KB) [file 12031_2024_2236_MOESM5_ESM.jpeg]

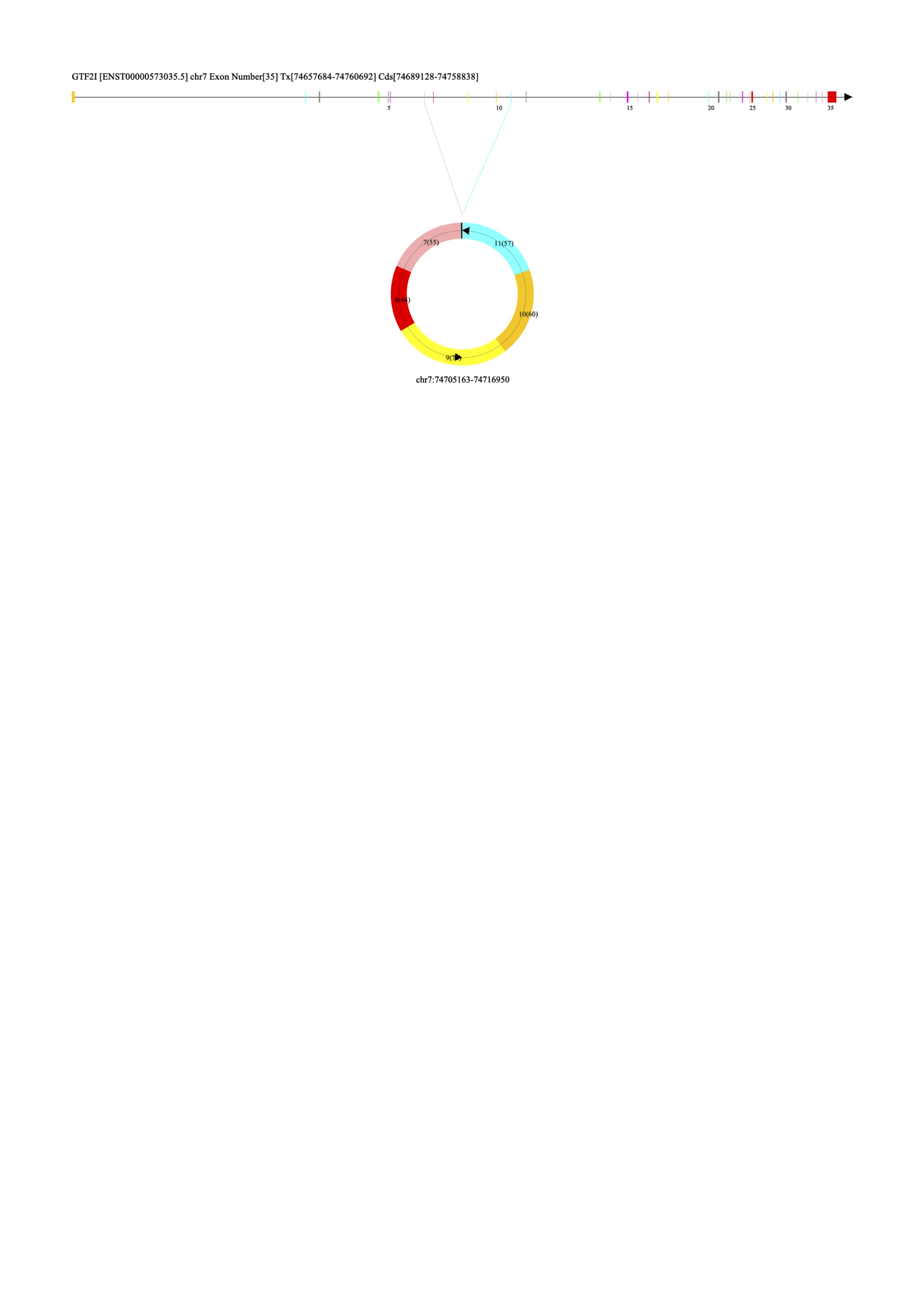

Supplement: Supplementary file 6 — Supplementary file6 (JPEG 185 KB) [file 12031_2024_2236_MOESM6_ESM.jpeg]

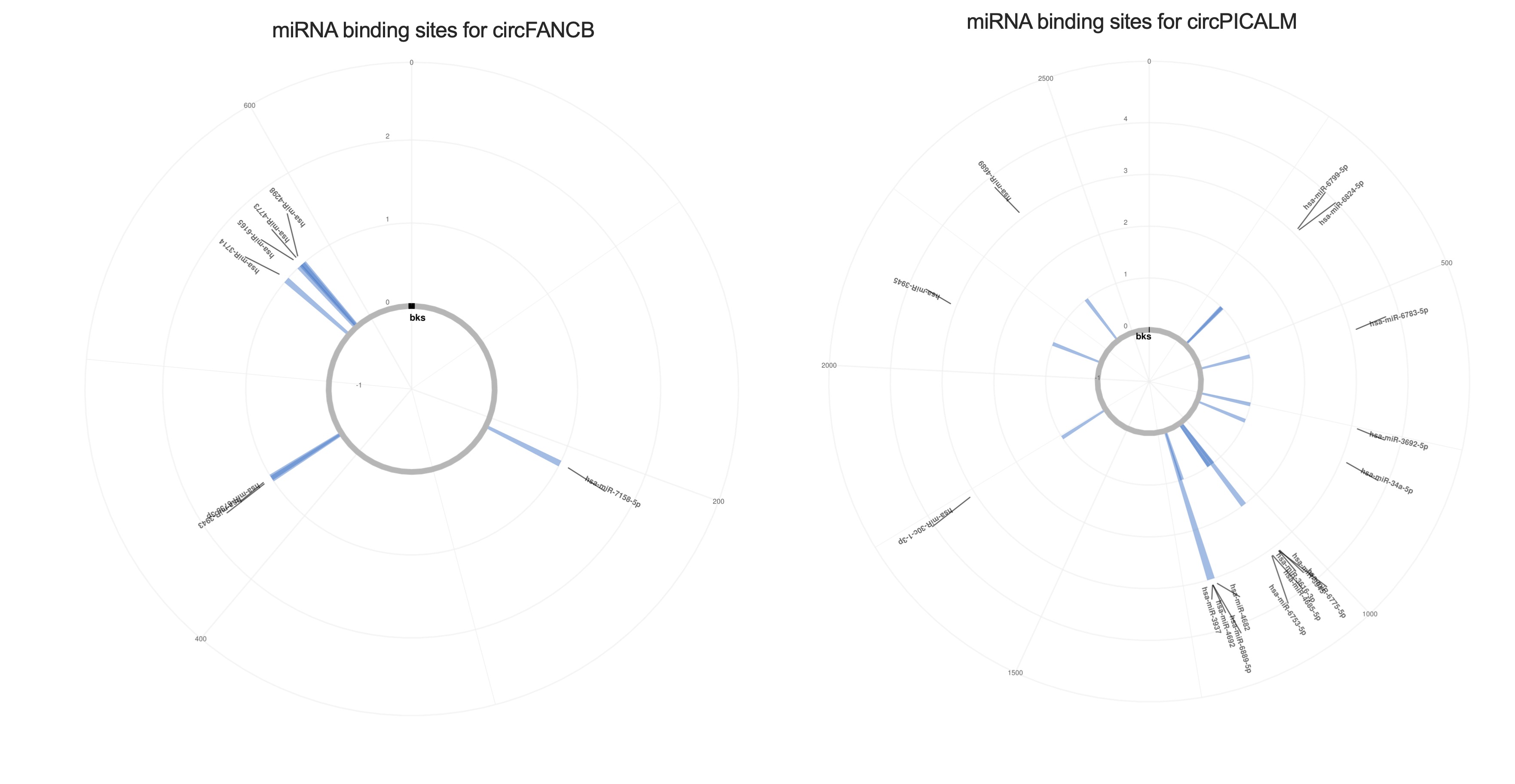

Supplement: Supplementary file 8 — Supplementary file8 (JPEG 269 KB) [file 12031_2024_2236_MOESM8_ESM.jpeg]

miRNA binding sites for circFANCB

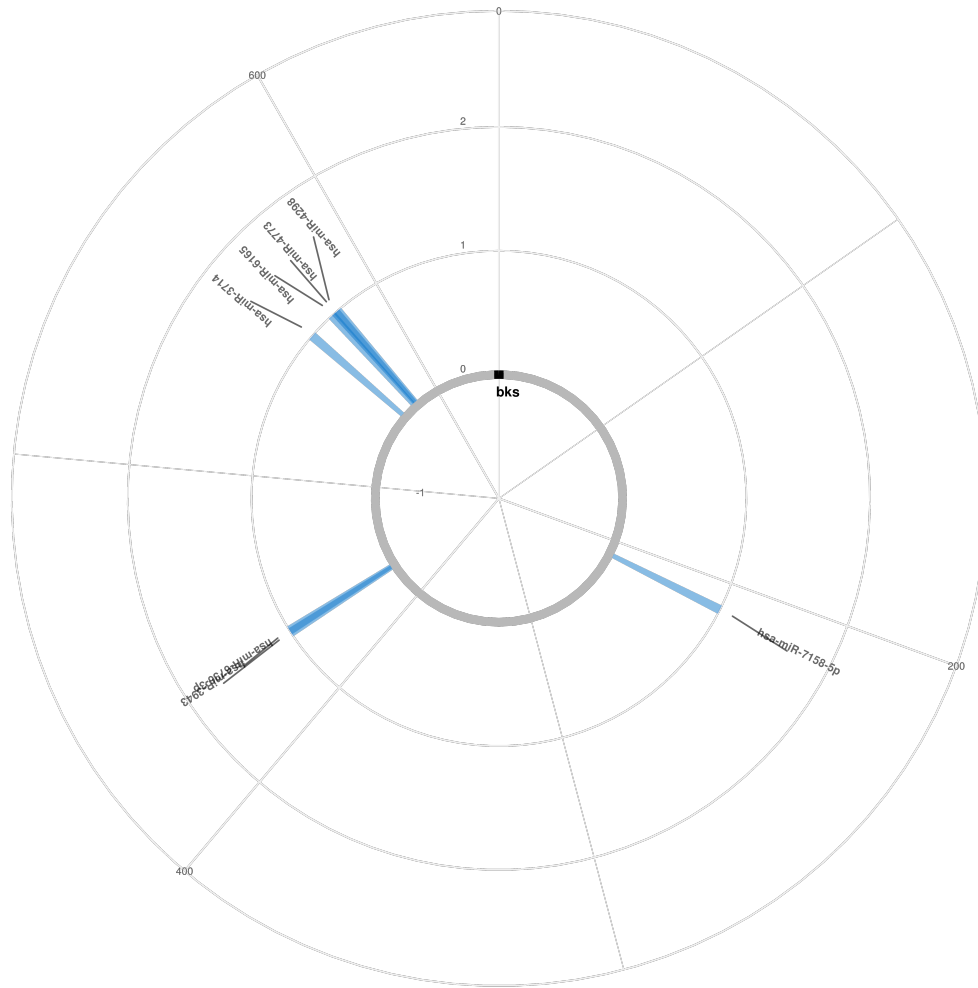

miRNA binding sites for circPICALM

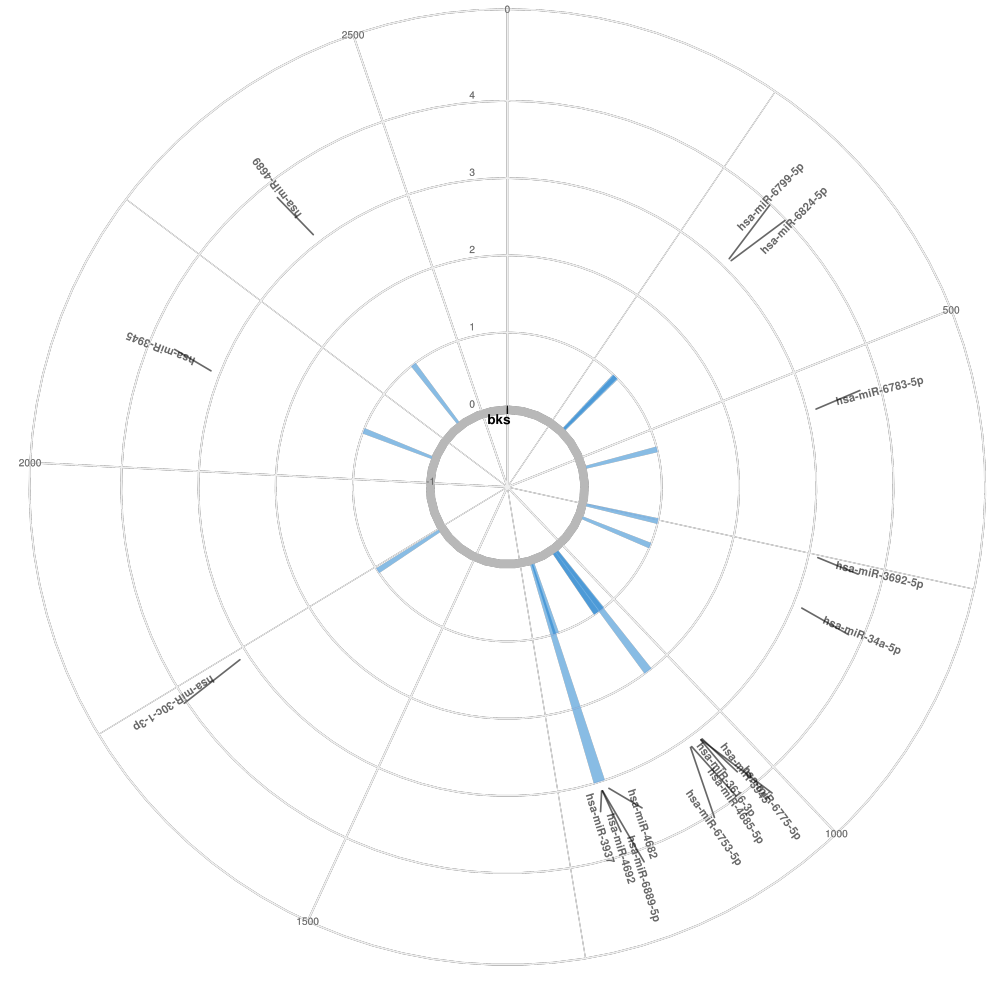

Supplement: Supplementary file 9 — Supplementary file9 (PDF 3313 KB) [file 12031_2024_2236_MOESM9_ESM.pdf]
