# Supplementary material for: Investigation of the Circular Transcriptome in Alzheimer’s Disease Brain
Source: J Mol Neurosci. 2024 Jul 9;74(3):64. doi: 10.1007/s12031-024-02236-0 (PMC11233389; doi:10.1007/s12031-024-02236-0)

ego2linear_up

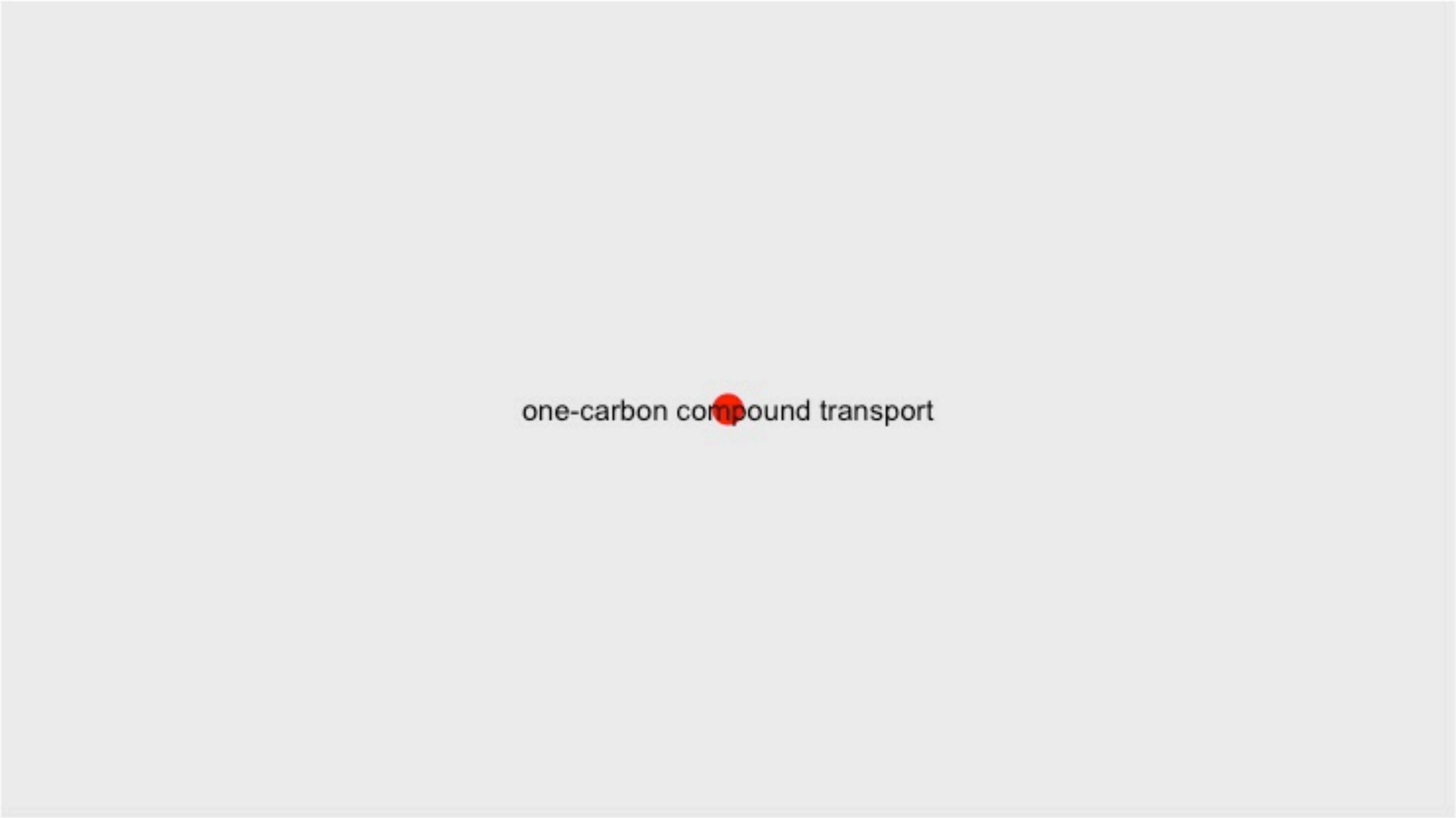

Supplement: Supplementary file 2 — Supplementary file2 (PDF 19 KB) [file 12031_2024_2236_MOESM2_ESM.pdf]
