# Supplementary material for: Investigation of the Circular Transcriptome in Alzheimer’s Disease Brain
Source: J Mol Neurosci. 2024 Jul 9;74(3):64. doi: 10.1007/s12031-024-02236-0 (PMC11233389; doi:10.1007/s12031-024-02236-0)
Supplement: Supplementary file 7 — Supplementary file7 (PDF 365 KB) [file 12031_2024_2236_MOESM7_ESM.pdf]

TFDP2 [ENST00000467072.5] chr3 Exon Number[14] Tx[141952480-142149532] Cds[141952512-142005443]

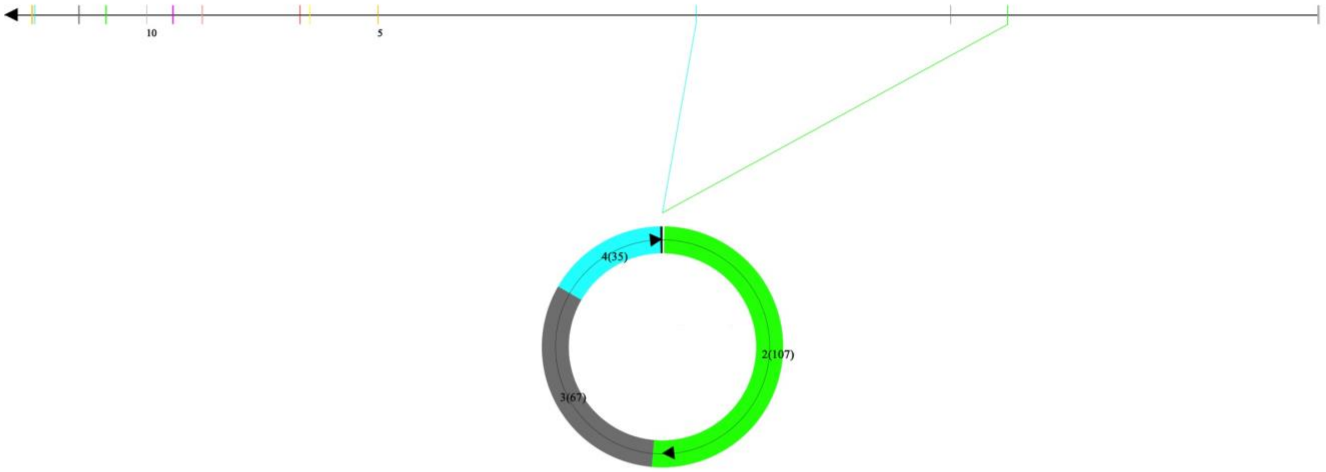

chr3:142054114-142101841

ATP13A3 [ENST00000256031.8] chr3 Exon Number[32] Tx[194402679-194468239] Cds[194405918-194462190]

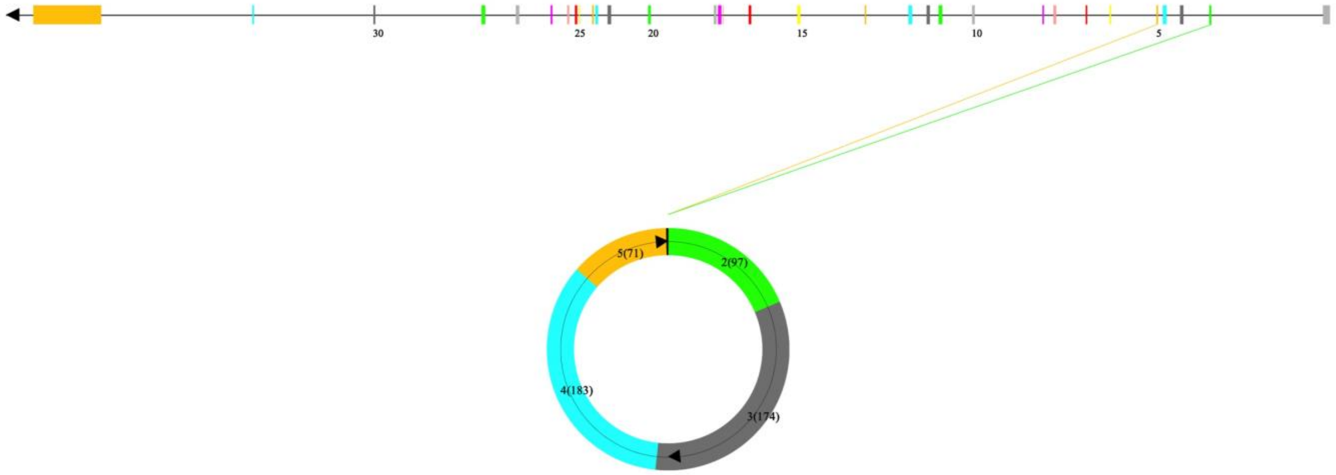

chr3:194459470-194462236

SEPT7 [ENST00000350320.10] chr7 Exon Number[13] Tx[35800985-35907105] Cds[35801209-35904293]

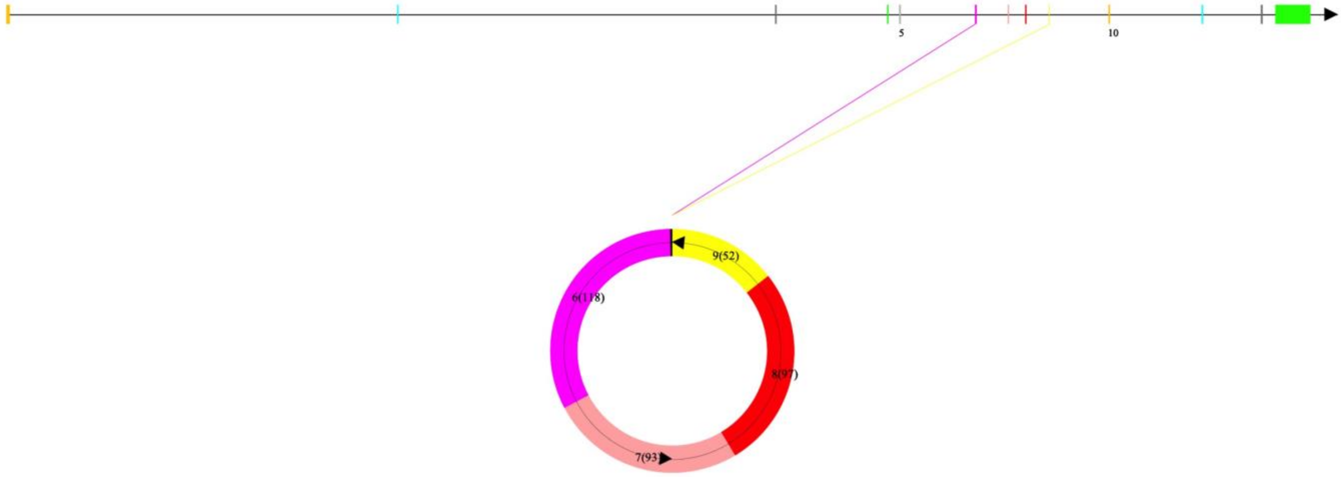

chr7:35879822-35885879

MCF2L2 [ENST00000328913.7] chr3 Exon Number[30] Tx[183178042-183428275] Cds[183179379-183427977]

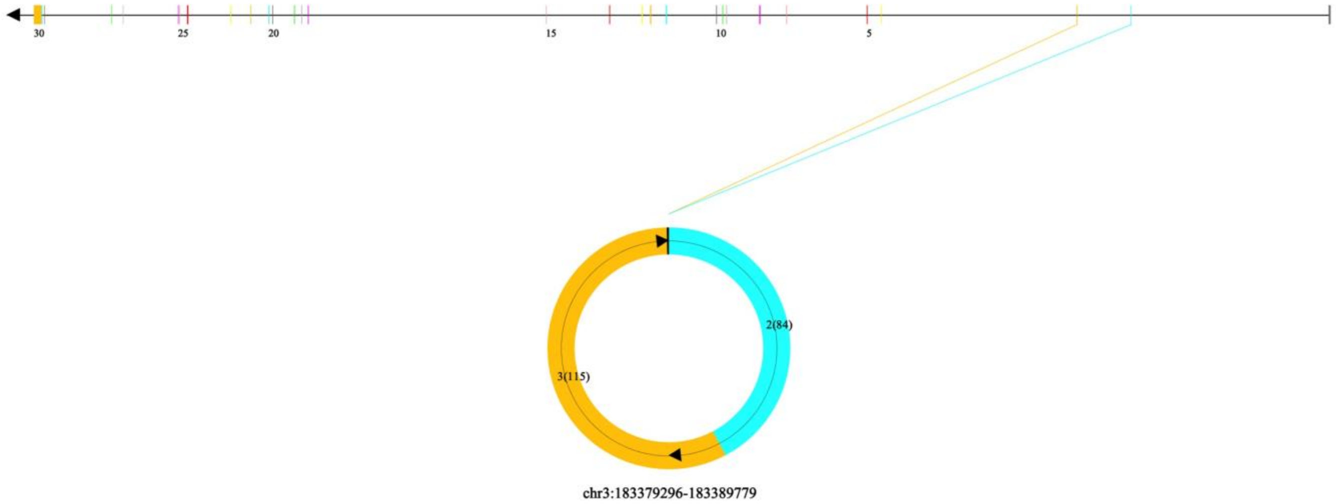

CCZ1B [ENST00000316731.12] chr7 Exon Number[15] Tx[6798360-6826770] Cds[6799223-6826197]

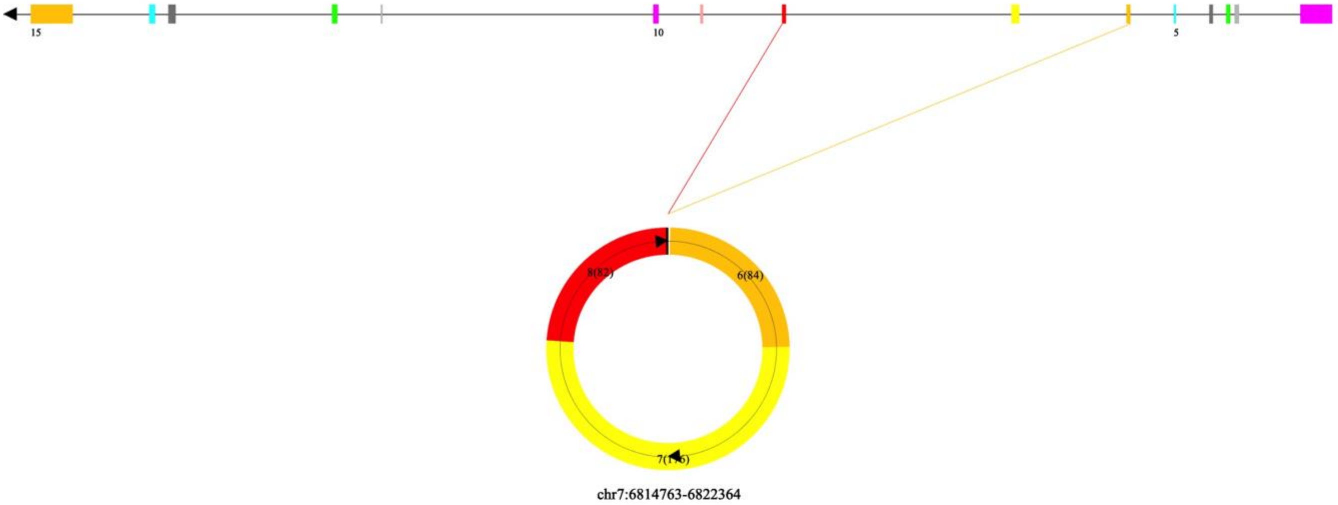

FANCB [ENST00000324138.7] chrX Exon Number[9] Tx[14843406-14873069] Cds[14843566-14865510]

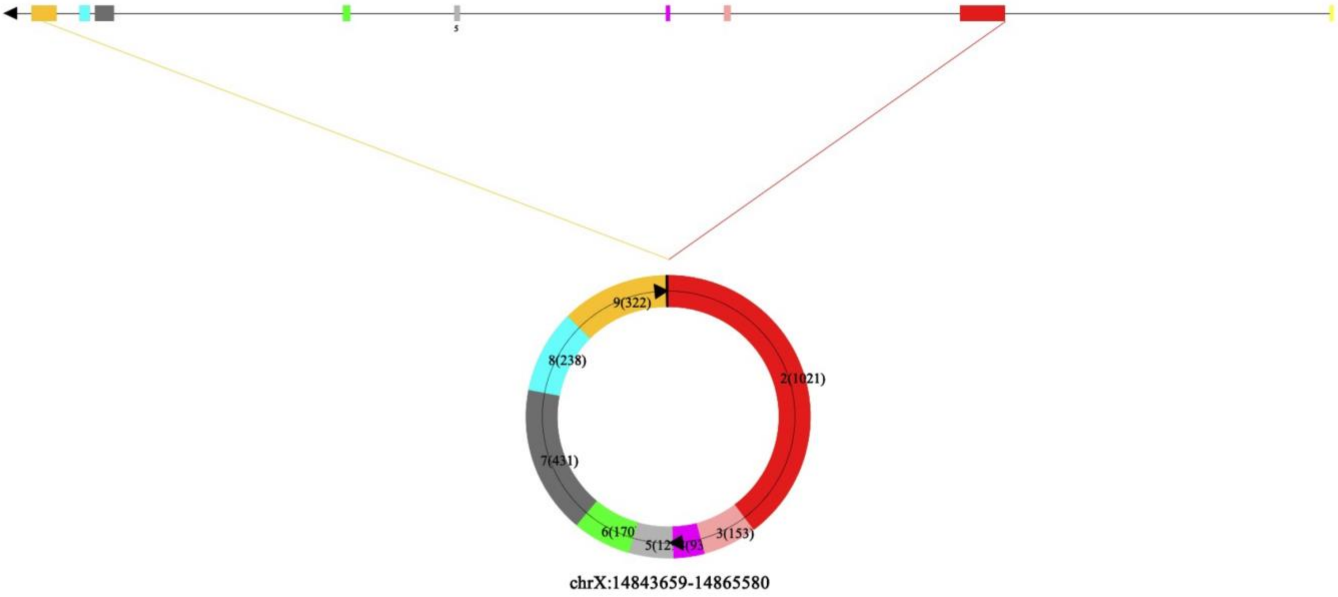

UBE4B [ENST00000253251.12] chr1 Exon Number[27] Tx[10032831-10180367] Cds[10033670-10179956]

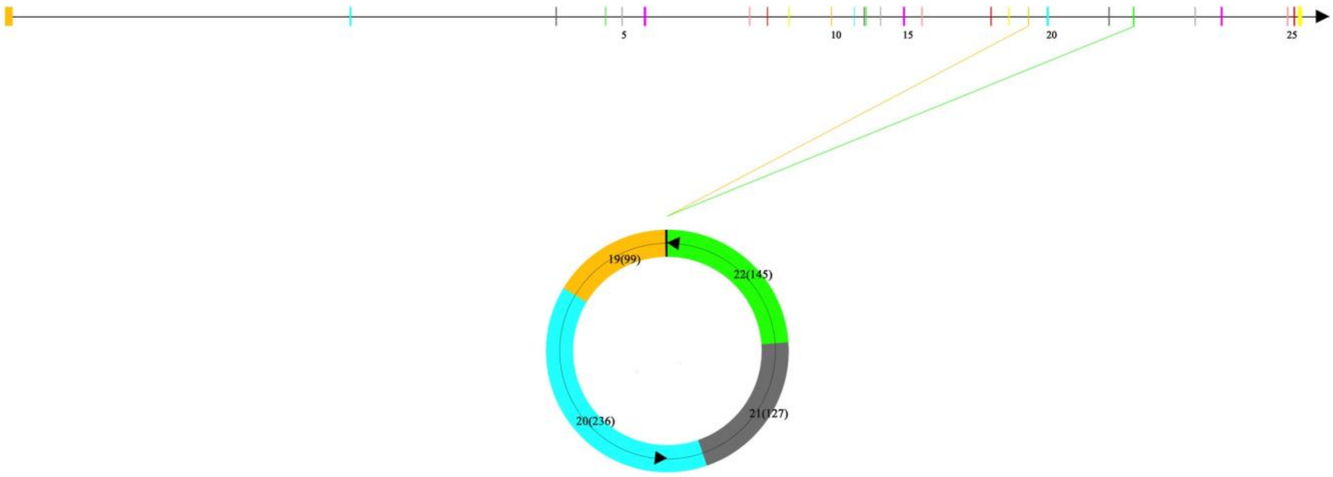

chr1:10149183-10161286

FASTKD1 [ENST00000453153.6] chr2 Exon Number[15] Tx[169529748-169573873] Cds[169529824-169572029]

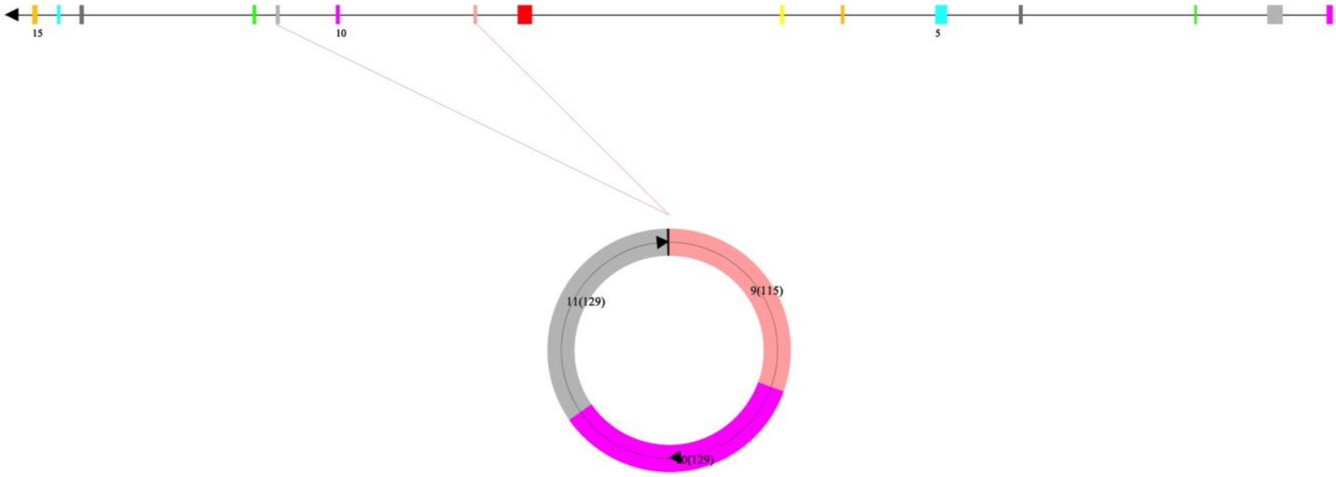

chr2:169538012-169544835

PICALM [ENST00000356360.9] chr11 Exon Number[19] Tx[85958986-86068780] Cds[85959045-86068780]

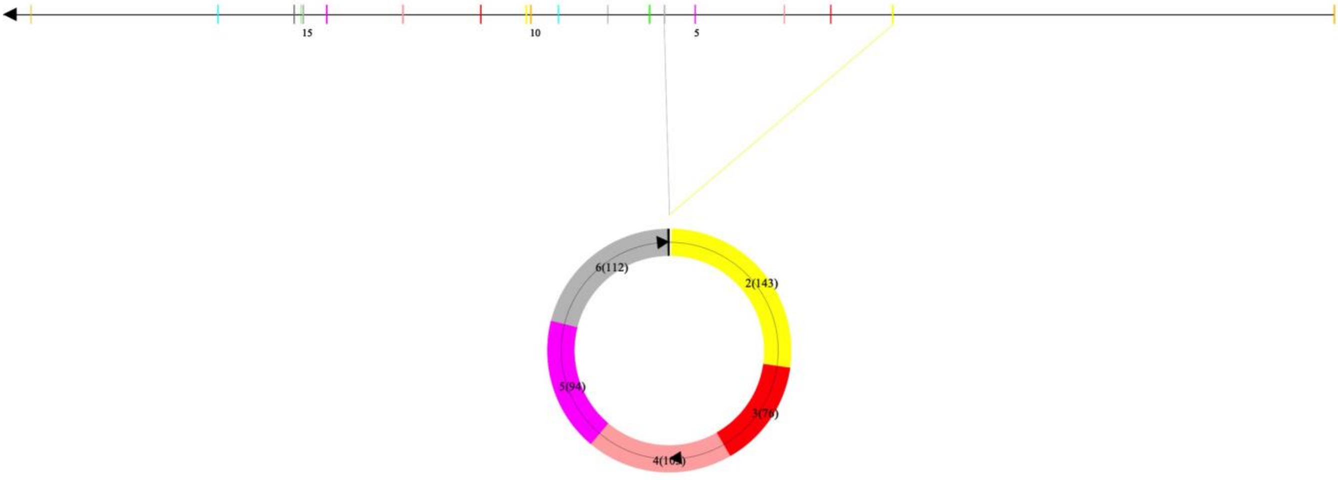

chr11:86012280-86031611

GTF2I [ENST00000573035.5] chr7 Exon Number[35] Tx[74657684-74760692] Cds[74689128-74758838]

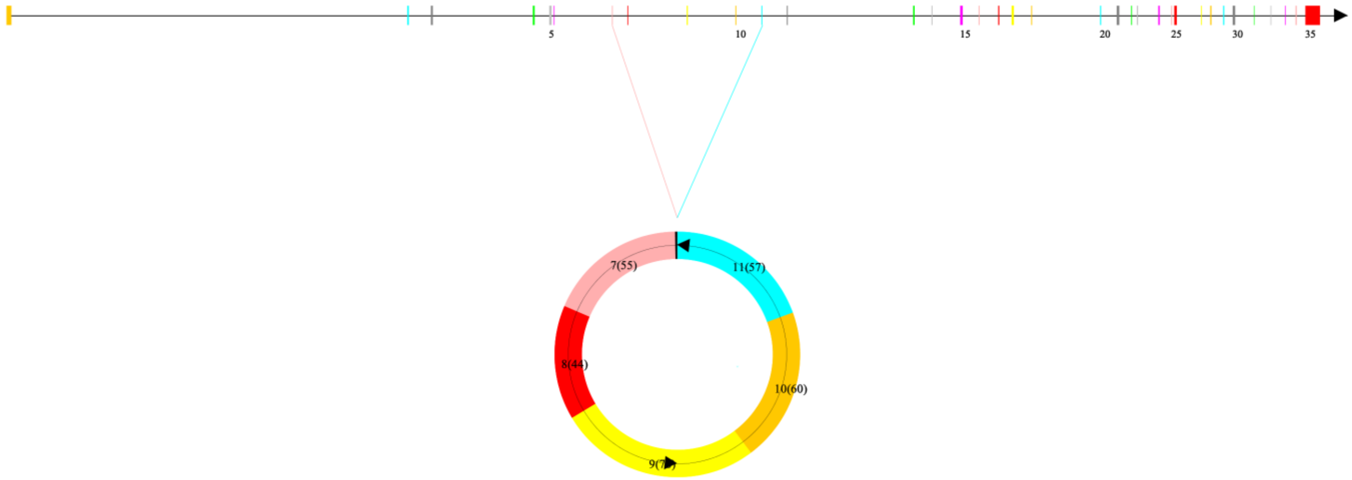

chr7:74705163-74716950
